# Glycan Fingerprint of Malignant Pleural Mesothelioma

**DOI:** 10.3390/ijms27146134

**Published:** 2026-07-09

**Authors:** Lovro Kavur, Thomas S. Klarić, Nikol Mraz, Nina Šimunić-Briški, Dora Lalić, Gordan Lauc, Martin Martinić, Lovorka Batelja Vuletić, Marina Martinić Kavur, Sven Seiwerth, Ozren Gamulin

**Affiliations:** 1 Clinical Department of Diagnostic and Interventional Radiology, Merkur University Hospital, 10000 Zagreb, Croatia; 2 Department of Biology, Faculty of Science, University of Zagreb, 10000 Zagreb, Croatia; 3 Genos Glycoscience Research Laboratory, 10000 Zagreb, Croatia; 4 Faculty of Pharmacy and Biochemistry, University of Zagreb, 10000 Zagreb, Croatia; 5 Faculty of Mechanical Engineering and Naval Architecture, University of Zagreb, 10000 Zagreb, Croatia; 6 Department of Pathology, School of Medicine, University of Zagreb, 10000 Zagreb, Croatia; 7 Department of Physics and Biophysics, School of Medicine, University of Zagreb, 10000 Zagreb, Croatia

**Keywords:** malignant pleural mesothelioma, glycosylation, N-glycans, FTIR spectroscopy, CNN classification, liquid chromatography, biomarkers

## Abstract

Malignant pleural mesothelioma (MPM) is an aggressive pleural tumor associated with asbestos exposure. Poor clinical outcome of MPM is often driven by late-stage diagnosis due to non-specific clinical presentation, similarity to pleural lesions (e.g., inflammatory changes, metastatic adenocarcinoma), and limitations of current diagnostic methods. We employed Fourier transform infrared (FTIR) spectroscopy combined with convolutional neural networks (CNNs) to analyze formalin-fixed paraffin-embedded (FFPE) pleural tissue samples from patients with MPM, metastatic adenocarcinoma, pleural inflammation, and normal (healthy) pleura. Glycan analysis of FFPE normal pleura and MPM was performed using ultra-high-performance liquid chromatography (UPLC) and mass spectrometry (MS). Our FTIR-spectral analysis uncovered a strong spectral fingerprint of MPM that was especially apparent in the region typical for C-O and C-C stretches as well as local symmetry region typical for deformation vibrations of CH_2_ and C-OH groups, all appearing in carbohydrates. Our orthogonal validation of these findings through a targeted glycomics approach using UPLC confirmed that the MPM N-glycome exhibits a distinct fingerprint that distinguishes it from normal pleural tissue. Through utilization of MS for identifying the exact structures of differentially expressed N-glycan peaks, we also identified two high-mannose N-glycan structures that show a specific biomarker potential for MPM and need to be examined in future studies.

## 1. Introduction

Malignant pleural mesothelioma (MPM) is an aggressive tumor primarily caused by asbestos exposure, with a latency period of approximately 40 years from exposure to symptom onset [1,2]. More than 60 countries banned asbestos use, resulting in peak incidence of MPM in the 1990s for some countries that introduced the ban (e.g., Norway, New Zealand, Germany) [3,4]. However, some highly populated countries, such as China, Brazil, Russia and Zimbabwe, continue to produce and export asbestos which is primarily used in India and China where incidence rates of MPM were predicted to increase between 2016 and 2030 [5,6]. MPM is notorious for its poor prognosis and relative resistance to conventional therapies. One major reason for treatment failure is late diagnosis—MPM is often advanced at presentation and can be misattributed to benign conditions [1,7,8]. Despite advances in imaging and molecular techniques, timely MPM treatment remains challenging due to the time-consuming, and sometimes inconclusive, diagnostic procedures currently available.

Traditional diagnostic approaches rely on cytological evaluation of pleural fluid and pleural biopsy, supplemented by immunohistochemistry using markers such as calretinin, cytokeratin 5/6, and more recently, BRCA-1 associated protein (BAP1) [9,10,11]. No single highly sensitive and specific biomarker for MPM exists in routine clinical use. Mesothelin (also known as soluble mesothelin-related peptide) is the most studied blood biomarker and is fairly specific, but its sensitivity is only ~30–50% (especially low in sarcomatoid subtypes and early-stage disease) [12]. Other candidates like fibulin-3 and osteopontin have not consistently outperformed mesothelin in clinical studies [12]. There is a compelling need for new approaches to facilitate earlier and more accurate diagnosis of MPM. A combination of rapid diagnostic tools that can differentiate MPM from other pleural conditions, and reliable early biomarkers could drastically improve patient prognosis.

N-linked glycans (N-glycans) are emerging as reliable biomarkers of numerous diseases, including cancer [13,14,15,16,17,18,19]. Several glycan and polysaccharide biomarkers have been identified in MPM so far. For example, bisecting *N*-acetylglucosamine (GlcNAc) at asparagine 388 (Asn388) of mesothelin is one of the main biomarkers for MPM in epithelioid mesothelioma and was identified by Fujihira et al. [20], the S1 monoclonal antibody that recognizes glycans with sulfate modifications which was identified as a diagnostic biomarker for MPM by Nakashima et al. [21], and the polysaccharide hyaluronic acid which has also been identified as a potential biomarker for MPM [22]. In addition, several proteins that have been identified as biomarkers for MPM, such as MUC1 [23] and Fibulin-3 [24] are known to be glycosylated. Moreover, Karunakaran et al. reported in their research paper looking at the MPM interactome that exposure to asbestos or asbestos fibers alters the transcription of *MGAT4A*, a gene that encodes a glycosyltransferase crucial for the synthesis of complex N-glycans, though the significance and the possible downstream effects of this observation were not explored [25]. The significance and diagnostic potential of glycans was acknowledged in the latest review about challenges in the pathological MPM diagnosis by Stefano et al. [26]. However, the application of glycomics in a clinical setting has been lagging behind other omics, in part due to the high level of technical skills and knowledge required for complex sample preparations and data interpretation [19].

Fourier transform infrared (FTIR) spectroscopy is a promising technique, offering the ability to detect molecular changes at the cellular level [27,28,29]. This technique measures vibrational modes of chemical bonds, providing a comprehensive molecular fingerprint of biological samples, but requiring no complex sample preparation procedures. Previous studies have demonstrated FTIR’s potential in distinguishing various cancer types, including prostate [30], ovarian [31], and hepatocellular carcinoma [32]. FTIR studies on the blood serum of MPM patients reported differences in lipid biosynthesis and nucleic acid levels; however, the biological basis of these spectral differences was not experimentally explored [33]. Recently, we published a FTIR study on pleural tissue samples that has shown promising results in differentiating MPM from other pleural conditions using principal component analysis (PCA) [34]. Here, we re-analyzed the full FTIR dataset from our recently published study [34] using a convolutional neural network (CNN) to identify the spectral region most discriminative for MPM among four pleural tissue types (MPM, pleural inflammation, metastatic adenocarcinoma, and normal pleura).

Our re-analysis of FTIR-spectra identified a fingerprint of MPM that was especially apparent in the region 900–1200 cm^−1^ (typical for C-O and C-C stretches and deformation vibrations of CH_2_ and C-OH groups of carbohydrates). We validated these findings in a different sample set (MPM and normal pleura) through a targeted glycomics approach using ultra-high-performance liquid chromatography (UPLC), and mass spectrometry (MS). Our results showed that extensive N-glycosylation changes distinguish MPM from normal pleural tissue and consist of high-mannose N-glycan structures that are not typically observed in other types of lung tumors or inflammation.

## 2. Results

### 2.1. CNN Classification Model

**Figure 1:**
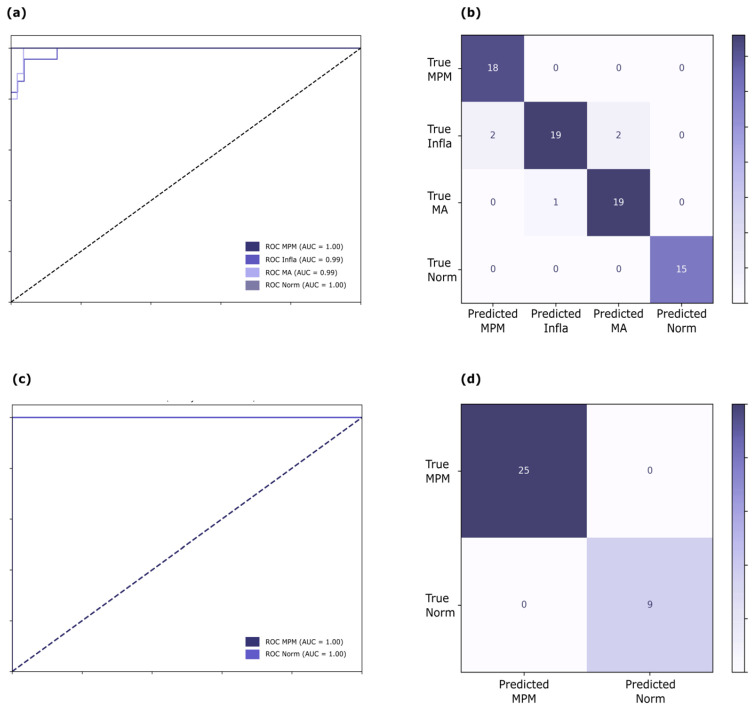
(**a**) ROC curve. The ROC curve shows the accuracy of the model for all four sample types (MPM, Infla = pleural inflammation, MA = metastatic adenocarcinoma, Norm = normal pleura). The Y-axis shows samples that were successfully classified into the correct category (true positive rate) while the X-axis shows samples that were incorrectly classified (false positive rate). (**b**) Confusion matrix. The Y-axis shows the pleural sample type (true label) while the X-axis shows the tissue type classification results using the CNN model (predicted label). (**c**) ROC curve for distinguishing MPM and normal pleura. The Y-axis shows samples that were successfully classified into the correct category (true positive rate) while the X-axis shows samples that were incorrectly classified (false positive rate). (**d**) Confusion matrix for distinguishing MPM from normal pleura. The Y-axis shows the actual pleural sample type (true label) while the X-axis shows the tissue type classification results using the CNN model (predicted label).

The CNN model achieved excellent performance in tissue classification with the following results: Test Accuracy: 93.42%, Precision: 93.61%, Sensitivity: 93.42%, and Specificity: 93.87%. The receiver operating characteristic (ROC) shows area under the curve (AUC) of 1 for normal pleura and MPM while pleural inflammation and adenocarcinoma metastasis have an AUC of 0.99 (Figure 1a). The confusion matrix demonstrated high accuracy across all tissue types. In two events, pleural inflammation was falsely classified as MPM (false positive), while the false negative rate for MPM was 0 (Figure 1b). When we restricted analysis only to MPM and normal pleura, AUC was 1 for both MPM and normal pleura (Figure 1c), and both the false positive and false negative rates were 0 (Figure 1d). Our model was also able to distinguish MPM from adenocarcinoma metastasis when challenged only with these two sample types, as well as MPM from pleural inflammation (Supplementary File S1).

### 2.2. Spectral Contribution to CNN Analysis

**Figure 2:**
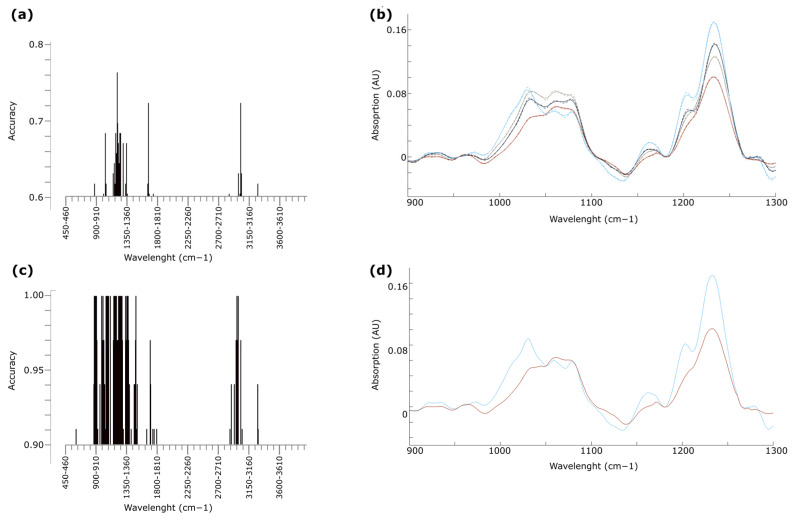
(**a**) Wavelengths with highest accuracy (model including all tissue types). On the X-axis are wavelengths (10 cm^−1^ steps), and on the Y-axis is plotted accuracy (cutoff at 60%). (**b**) FTIR-spectra. On the X-axis are wavelengths (900–1300 cm^−1^) and on the Y-axis absorption. All tissue types are shown: normal pleura (light blue), pleural inflammation (teal), adenocarcinoma metastases (beige) and MPM (red). (**c**) Wavelengths with highest accuracy (model including only normal pleura and MPM). On the X-axis are wavelengths (10 cm^−1^ steps), and on Y-axis is plotted accuracy (cutoff at 90%). (**d**) FTIR-spectra. On the X-axis are wavelengths (900–1300 cm^−1^) and on the Y-axis absorption. Only normal pleura (light blue) and MPM (red) are shown.

FTIR analysis revealed distinct spectral differences between the four tissue types across multiple regions, and the model for tissue identification was performed by taking the entire imaged spectra into consideration. However, the contribution was not equal among all regions. To break down the spectra to areas with the highest contribution, we grouped the measured wavelengths to 50 cm^−1^ steps (Table 1) and 10 cm^−1^ steps (Figure 2a,c; Supplementary Files S2 and S3). The highest accuracy of the CNN model was observed in the region typical for the C-O and C-C stretches as well as deformation vibrations of CH_2_ and C-OH groups appearing in carbohydrates (Table 1 and Figure 2a,c). The differences between vibrational patterns of different tissue types in this region are visible on the FTIR-spectra (Figure 2b,d).

MPM shows a distinct FTIR-spectral fingerprint in the 900–1300 cm^−1^ region that differentiates it from all other analyzed tissue types, pointing to the fact that this region could contain molecular signatures that are different in all 4 examined pleural tissue types (Figure 2b). The difference is especially apparent in the comparison between normal pleura and MPM (Figure 2d) This region is considered biologically interesting due to the fact that many carbohydrate molecules, e.g., polysaccharides and glycans, absorb in this region while proteins, which typically dominate the FTIR-spectra in terms of absorption, do not contribute significantly to this region [35,36]. Moreover, Derenne et al. showed that FTIR can be used for the analysis of purified glycoproteins and that absorption in the region 1000–1200 cm^−1^ is not only sensitive to drastic differences (such as the presence or absence of multiple monosaccharides), but also to subtler changes, such as small glycan modifications [35,37]. However, in comparison with purified glycoproteins, tissue samples of pleura are extremely complex and multiple issues can arise in the FTIR analysis of complex biological samples as highlighted by Eissa et al. [38]. Therefore, independent methods need to be used to verify the assumption that the observed differences are due to differences in the carbohydrate composition of the tissues. As UPLC was previously combined with FTIR for in-depth glycosylation analysis [39], we decided to take the same approach to compare the N-glycomes of normal pleura and MPM.

### 2.3. N-Glycomics Analysis of Human Pleural Tissue

**Figure 3:**
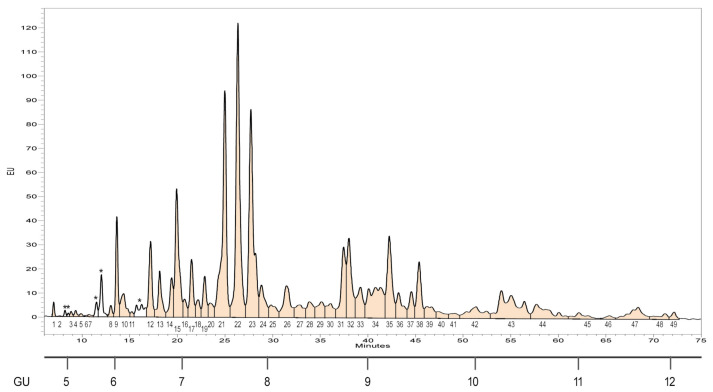
Representative chromatogram of N-glycans derived from FFPE of normal human pleura. Integration boundaries between N-glycan peaks are shown as vertical lines (49 peaks). The X-axis displays analyte retention time, while the Y-axis shows fluorescence at 370 nm. Peaks indicated with an asterisk (*) overlap with free oligosaccharides and hence were not included in subsequent analyses. GU—glucose units, EU—emission units.

Having identified the carbohydrate absorption region (900–1200 cm^−1^) as the most discriminative spectral feature for MPM by FTIR, we next sought to validate this finding at the molecular level using orthogonal glycan analysis. Due to the preexisting N-glycome data for lung adenocarcinoma and inflammation available in the literature, and the fact that MPM N-glycomics remains largely uncharacterized, this validation was focused specifically on MPM versus normal pleura. We analyzed archival FFPE tissue from two groups: normal pleura (n = 30 samples, from 30 individuals) and MPM (n = 30 samples, from 22 patients). N-glycans were released from FFPE sections, labeled with procainamide (ProA), and subjected to linkage-specific derivatization of N-acetylneuraminic acid (Neu5Ac) (Supplementary File S4). UPLC profiling identified 49 distinct N-glycan peaks in both normal pleura and MPM samples. (Figure 3, Supplementary File S5). All individual samples shared the same profile characteristics, but MPM samples showed higher inter-individual variation than controls (Supplementary File S6).

**Figure 4:**
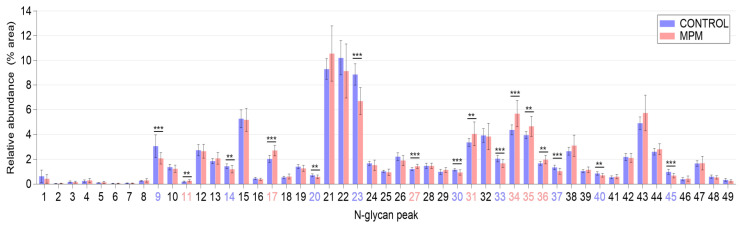
Normalized mean relative abundance of each N-glycan peak in the pleura N-glycome. Error bars show the standard deviation. Multiple unpaired *t* tests with Welch correction were used to statistically determine the significant differences in N-glycan peak abundance between groups and these peaks are marked with asterisks. *p* values (after correction for multiple testing) are as indicated: ** *p* < 0.01, *** *p* < 0.001. N-glycan peak labels in red font indicate that the peak is more abundant in MPM than in the control group, while blue font indicates that the peak is less abundant in MPM.

However, the relative abundance of several N-glycan structures differed significantly between groups. Out of 49 N-glycan peaks, 16 peaks (33% of the total number of peaks) were significantly different between the two groups (Figure 4). Notably, the abundance of peaks 9, 14, 20, 23, 30, 33, 37, 40 and 45 was significantly lower in the MPM group, while the abundance of peaks 11, 17, 27, 31, 34, 35 and 36 was significantly lower in the control group (Figure 4).

**Figure 5:**
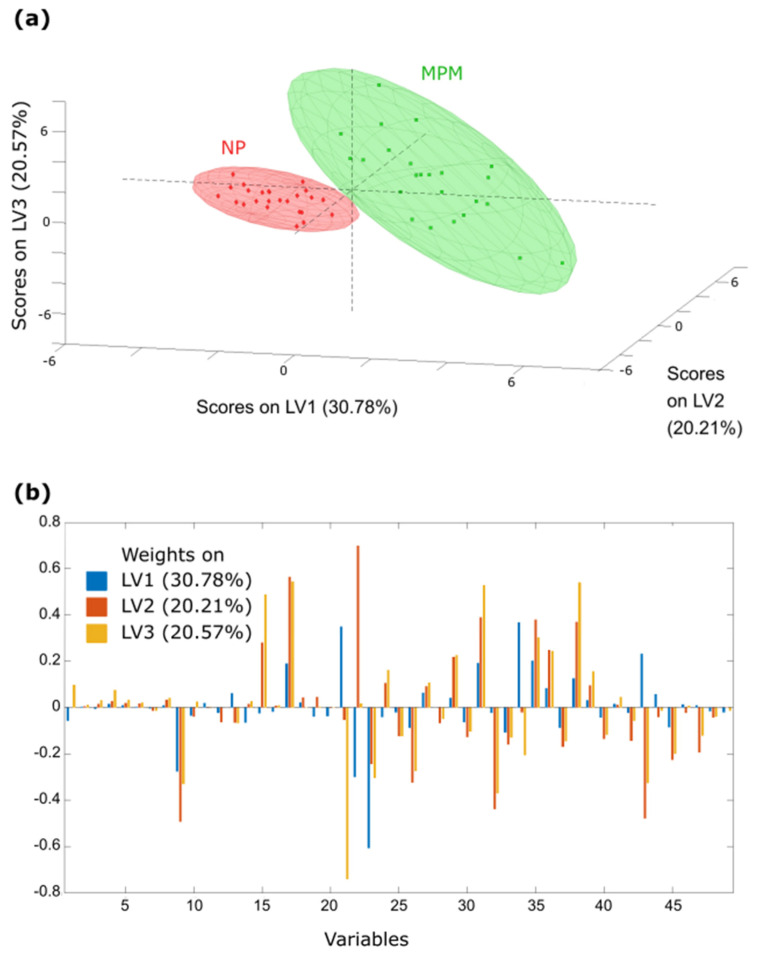
PLS-DA analysis of the UPLC data. (**a**) The relationship between the control pleura samples (red) and MPM samples (green) at the level of the total N-glycome was visualized using a PLS-DA. The X-axis, Y-axis and Z-axis display the Level 1–Level 3 (LV1, LV2, LV3). (**b**) Contribution (weights) of variables to LV1-3 in PLS-DA analysis. Weights are plotted on Y-axis and variables (UPLC glycan peaks 1–49) on the X-axis. LV1 = blue, LV2 = red, LV3 = Yellow.

Furthermore, to check whether MPM and normal pleura can indeed be distinguished at the level of the total N-glycome, we performed partial least squares analysis (PLS-DA). PLS-DA of N-glycan peak abundance data showed the clear segregation of control and MPM samples in 3D separation (Figure 5a); the weights of individual peaks used for each of the three levels is shown in Figure 5b.

### 2.4. Identification of Differentially Expressed N-Glycan Structures

**Figure 6:**
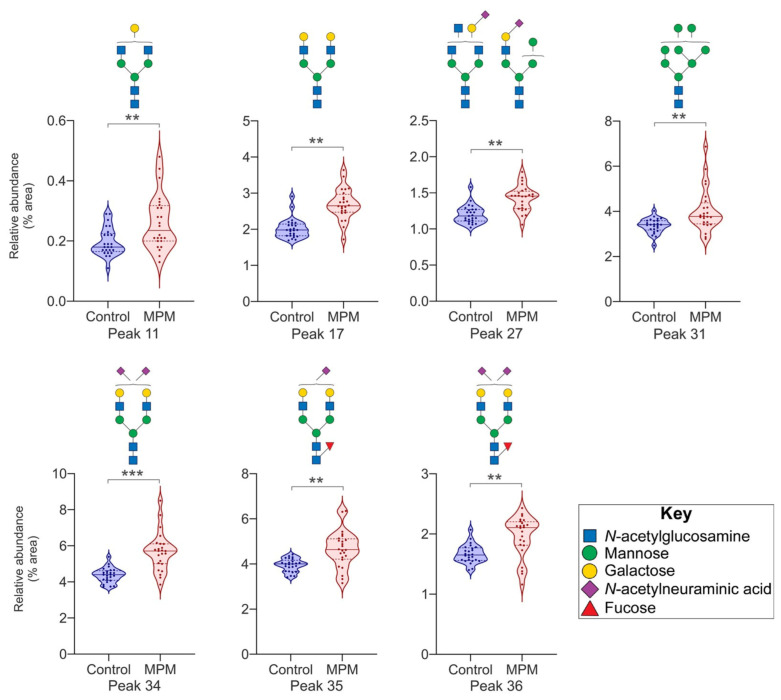
N-glycan peaks whose abundance is significantly increased in MPM. Violin plots show the spread of peak abundance within each group for the given glycan peaks. The mean value is indicated by the solid horizontal line, while the quartile values are indicated by dotted horizontal lines. The proposed structure of the major N-glycan within each peak is illustrated above the violin plots. Where the composition of the major N-glycan differs between groups, both structures are illustrated, each above their respective violin plots. ** 0.001 < *p* < 0.01, *** *p* < 0.001.

To identify the exact N-glycan structures eluting in the UPLC peaks that differed significantly between control samples (normal pleura) and MPM, we performed additional structural analysis using mass spectrometry (MS) (Supplementary File S4). Out of 16 differentially expressed N-glycan peaks, majority (13/16) contained the same dominant N-glycan structure in both control and MPM (Supplementary Files S7 and S8). Six peaks that were upregulated in MPM were mono- or di-galactosylated complex bi-antennary N-glycan structures with or without core-fucose and/or sialic acid, while only one was a high-mannose structure (Figure 6). In Peak 27 the major glycan structure differed between normal pleura and MPM.

**Figure 7:**
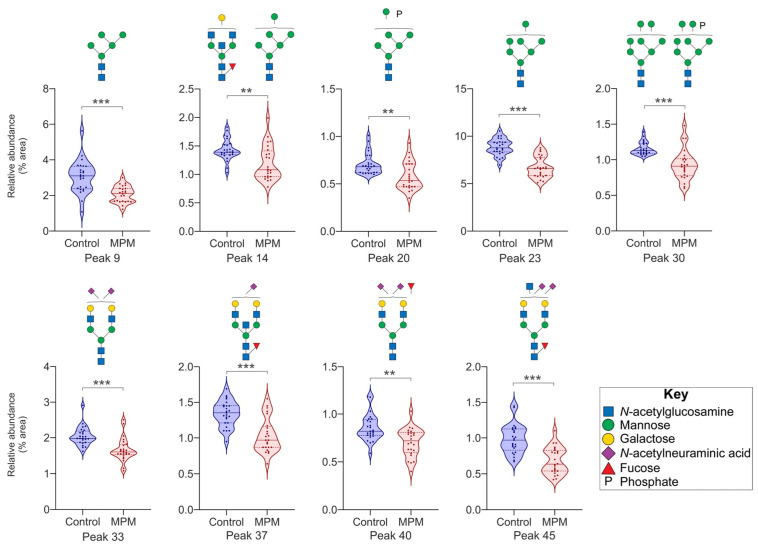
N-glycan peaks whose abundance is significantly decreased in MPM. Violin plots show the spread of peak abundance within each group for the given glycan peaks. The mean value is indicated by the solid horizontal line, while the quartile values are indicated by dotted horizontal lines. The proposed structure of the major N-glycan within each peak is illustrated above the violin plots. Where the composition of the major N-glycan differs between groups, both structures are illustrated, each above their respective violin plots. ** 0.001 < *p* < 0.01, *** *p* < 0.001.

Out of nine N-glycan peaks that were significantly downregulated in MPM, two minor peaks (Peak 14 and Peak 30) again contained different dominant structures in control and MPM samples, while seven peaks had the same main structure in both control sample and MPM. Out of those seven peaks, three were high-mannose structures while the remaining four peaks were sialylated complex bi-antennary glycan structures with or without core fucose (Figure 7).

## 3. Discussion

This study demonstrates that FTIR spectroscopy combined with CNN can effectively differentiate MPM from other pleural conditions, with the most discriminative spectral features located in the carbohydrate absorption region (950–1200 cm^−1^). The CNN model’s high accuracy (93.42%) demonstrates future clinical potential if the results are replicated in a larger study. The spectral region between 1150 and 1200 cm^−1^ alone achieved 97.37% accuracy, suggesting that targeted analysis of glycan-related spectral features could potentially serve as a biomarker for MPM. Previous FTIR studies have shown promise for MPM diagnosis [40], and differentiation from other benign and malignant pleural states [34], but this is the first study that identified the carbohydrate absorption region as a key contributor to this differentiation. Nevertheless, recent methodological studies warned about the pitfalls of overly intensive molecular interpretation of FTIR-spectra of complex biological samples. Eissa et al. (2023) emphasize that although FTIR can reliably distinguish samples using machine learning, caution is needed when attributing a specific spectroscopic difference to a precisely determined molecule, due to possible overlaps [38].

In this study, ambiguity was minimized by combining FTIR with orthogonal glycan analysis (UPLC, MS), whereby we confirmed that spectral changes indeed correlate with changes in the N-glycan profile and identified the structures of potential N-glycan culprits responsible for the observed spectral changes. Voronina et al. previously showed that a combination of FTIR and UPLC methods is compatible for in-depth glycome analysis of complex samples with large numbers of different biomolecules, such as blood plasma [39]. By using orthogonal analytical methods we were able to confirm that the spectral differences observed by FTIR reflect genuine molecular changes visible in a distinct N-glycosylation fingerprint of MPM.

Glycosylation changes in tumors are not unexpected. In fact they appear to play a role in extracellular signaling, tumor cell migration, modulation of tumor growth, tumor invasion into other tissues, immune response to tumors, etc., and are consistently present in many different tumor types [15,41,42]. Several studies performed N-glycome analysis from FF and FFPE tissues of various lung carcinomas including lung adenocarcinoma [43], non-small-cell lung cancer [18], small-cell lung cancer [44] and others [17]. Additionally, Fujihira et al. (2021) investigated epithelial MPM and discovered that mesothelin carries a specific N-glycan modification, bisecting GlcNAc at one of its N-glycosylation sites (Asn388) which mesothelin in normal cells does not contain [20]. However, this is the first study that identified extensive changes across the MPM N-glycome which suggests that the changes are more widespread than a single glycoprotein and could affect the proteome at large.

Our UPLC analysis identified 49 N-glycan peaks in both normal pleura and MPM tissue. This number is comparable to previously published lung N-glycome by Wang et al. who identified 58 N-glycan peaks in FFPE tissue sections of human lung [45], and Lattova et al. who identified 52 glycan peaks in fresh and snap-frozen lung tissue [46]. We followed up on the UPLC analysis by performing MS of differentially expressed N-glycan peaks. The structures that we identified using MS were previously reported as a part of normal lung N-glycome in FFPE tissue sections [45], frozen samples [47] and fresh lung tissue [48]. The process of tissue fixation does not affect N-glycome if performed promptly after sampling [49,50]. Jia et al. characterized the lung glycome in detail and found that at least 75% of the lung glycans carried sialic acid, only 30% of lung glycans were fucosylated, and only 5% of all lung glycans had bisecting GlcNAc, but 80% of those bisected structures were fucosylated and only 25% sialylated, while phosphorylated N-glycans represented only a minor component [51]. Although we did not identify the glycan structures within all detected N-glycan peaks in our current study, it is worth noting that out of 16 differentially expressed N-glycan peaks, 5 peaks were high-mannose glycans, and 11 were complex N-glycans (5 fucosylated complex N-glycans, 8 sialylated complex N-glycans, and 2 bisected complex N-glycans, with both of the bisected N-glycans carrying core fucosylation). One differentially expressed glycan peak in the control group (6% of all differentially expressed N-glycan peaks) and two differentially expressed peaks in MPM (12.5%) were phosphorylated high-mannose N-glycans.

Interestingly, high-mannose N-glycans are typically increased in lung inflammation and cancer [46], a pattern that was not present in differentially expressed N-glycan peaks within MPM tissue examined in our study. In particular, UPLC peak 23, a prominent peak with dominant N-glycan structure H7N2 (Figure 7), was significantly decreased in MPM in comparison to normal pleura (6.7% vs 8.9% of the total N-glycome, respectively). This represents a significant finding for distinguishing MPM from lung adenocarcinoma metastases, since both Ruhaak et al. (2015) and Lattova et al. (2020) identified the same glycan structure (H7N2) as significantly increased in lung adenocarcinoma when compared to the non-cancerous tissue [47,48]. In a follow-up study, Lattova et al. also identified an increase in high-mannose N-glycans (H6-9N2) as a distinct feature of lung inflammation and different lung cancer types, including lung adenocarcinoma, squamous carcinoma, and small-cell lung cancer [46]. Additionally, most previous studies report no difference in the expression of H5N2 between cancerous and non-cancerous lung tissue [46,47,48], and Lattova et al. attributed this difference between H5N2 and H6-9N2 levels in lung cancer to the expression levels of Golgi mannosidase *MAN1A1* that affects H6-9N2 but not H5N2. In contrast, in our results, H5N2 is significantly lower in MPM than in control tissue (Figure 7 and Table 2). Decreased levels of high-mannose glycans were previously observed in the stratum corneum of aging human skin [52] and in ageing murine epidermal cells where they were again linked with upregulation of the *Man1a* gene [53]. Though lung pleura is a serous membrane consisting of only a monolayer of mesothelial cells, both pleura and skin are protective barriers composed of flattened cells that are exposed to friction and other negative effects over time. Nothing is currently known about the effects of ageing on N-glycosylation in human lung pleura, but it is possible that the same principal observed in skin also applies to pleura, i.e., that ageing decreases levels of high-mannose glycans. It would therefore be important to carefully age-match the cases and controls in future studies.

The expression level of other high-mannose glycans in our study is not entirely clear as there appears to be a shift in isoforms and/or secondary modifications (e.g., phosphorylation) between control and MPM tissues rather than the general change in the expression of a specific isoform, as observed for the peaks 14 and 20 (H6N2) as well as peaks 30 and 31 (H8N2). However, further studies need to be made to determine whether isomer/secondary modification changes are a feature of MPM and/or supported by other changes in glycosylation machinery.

Results of complex N-glycans that are differentially expressed between MPM and control tissue of normal pleura are more difficult to interpret as the changes observed in the literature are not entirely consistent. For instance, H4N4 is increased in MPM and though it was not reported as significantly changed in lung adenocarcinoma by Ruhaak et al. or Lattova et al. (2020) in progressive lung adenocarcinoma stages, it was somewhat increased in different types of lung cancer and inflammation in their more recent publication [46,47,48]. H5N4 is also significantly increased in MPM, and its expression was previously reported as significantly lower in adenocarcinoma by Ruhaak et al. and decreased in progressive lung adenocarcinoma stages by Lattova et al., though the latest Lattova et al. study also lists it as increased in several lung cancer types as well as inflammation [46,47,48]. Sialylated glycans, such as H5N4F1S1 which are increased in MPM (Figure 6), are reported as mostly decreased in Ruhaak et al., but not Lattova et al. [46,47,48]. It should be noted, however, that many N-glycan structures show a distinct spatial distribution even within healthy human lungs, and the inconsistencies between previous studies in the literature could be the result of the difference in tissue sampling [54].

Despite promising research results, vibrational spectroscopy remains in the research phase due to the lack of standardization and validation in large clinical studies [27,30,55]. Since FTIR does not require sample preparations such as fluorescent labeling or purification, it could be at present useful as a screening method of choice for discovering biological samples with high biomarker potential. The advantages to that approach could be the availability of pre-recorded FTIR-spectra for re-analysis with machine learning algorithms and samples deemed as having “high potential for biomarker discovery”. But for any future use of FTIR-spectroscopy in routine clinical diagnostics, high reproducibility also needs to be ensured as different sample preparation and recording protocols can result in variabilities in spectra [56]. We also note that the current CNN implementation does not include probability calibration or early stopping based on a held-out validation partition; incorporating these refinements in larger follow-up studies would be advisable before venturing into clinical translation of this approach. Additionally, FTIR spectra need to be combined with orthogonal methods such UPLC and MS to gain deeper biological understanding of underlying molecular changes, as demonstrated by this study.

Our present study is limited due to the relatively small sample size and would need to be validated in larger cohorts. However, due to the difficulties in obtaining samples (especially rare cancer types), a sample size of 20–100 samples is standard practice. Despite the relatively small sample number with a high risk of overfitting, we were able to achieve two major break-throughs in molecular characterization of MPM. First, through the use of FTIR data and a CNN model, we were able to pin-point the spectral region attributed to glycans as highly accurate and specific for distinguishing MPM from other pleural pathologies (Figure 1 and Figure 2 and Table 1). Second, through orthogonal validation by UPLC and MS we were able for the first time to identify a distinct N-glycan fingerprint of MPM that differentiates it from normal pleura (Figure 3, Figure 4, Figure 5, Figure 6 and Figure 7) and previously reported N-glycan profiles of other lung cancers and inflammation (Table 2). Two prominent peaks containing high-mannose N-glycans from our study: peak 9 with the dominant structure H5N2 and peak 23 with the dominant structure H7N2 are highly significantly decreased in MPM while previous studies report either no significant change (H5N2) or increase (H7N2) in adenocarcinoma and inflammation (Table 2). Two additional high-mannose glycans (H6N2, H8N2) that were consistently reported as increased in the previous literature covering different types of lung cancer and inflammation show a pattern of isomer switching and/or phosphorylation change in the present study (Table 2).

Our finding, if replicated, could provide a partial explanation for why adenocarcinoma metastases and pleural inflammation group closer together in the PCA (Sadiku-Zehri et al.) since high-mannose glycans and their different isomers participate in the recruitment and response of cells and molecules involved in innate and adaptive immunity [34,57,58]. High-mannose glycans are also a frequent substrate for the Mannose-6-phosphate pathway (M6P) that requires mannose phosphorylation to transport N-glycosylated enzymes to lysosomes, organelles crucial for immune function, cellular remodeling and waste breakdown [59,60]. Among previously suggested MPM biomarkers, Cathepsin D is a lysosomal protease that affects tumor progression and metastasis [61,62,63] and it was also suggested as a biomarker of tissue aging [64]. However, though this connection with previously suggested biomarkers is worth noting, due to the extent of the N-glycosylation changes observed in our study it is much more likely that multiple glycoproteins are affected. Whether these N-glycome changes are related to tissue ageing (potentially due to the asbestos exposure) and/or somehow connected to lysosomal dysfunction remains to be explored.

Distinct N-glycan fingerprint of MPM discovered in this study and characterized by atypical changes in high-mannose glycans presents a significant biomarker potential and opens new avenues for future research of MPM.

## 4. Materials and Methods

This study was approved by the Ethics Committee of the School of Medicine, University of Zagreb, code number: 380-9-10106-19-111/222, class: 641-01/19-02/01, 18 September 2019. All samples were coded to ensure patient anonymity, with identifying information stored separately with restricted access.

### 4.1. FTIR Data Processing and Convolutional Neural Network Model

An entire dataset of spectral data recorded in the study by Sadiku Zehri et al. [34] was re-processed using MATLAB 2021a with PLS Toolbox 9.0. All available patient information and FTIR analysis methodology is provided in Sadiku Zehri et al. published in 2020. Briefly, FFPE tissue samples of 32 patients with diagnosis of MPM, pleural inflammation, metastatic adenocarcinoma (10 per class) or normal pleura (2) were obtained from the Department of Pathology archives (University of Zagreb, School of Medicine) and sectioned to 3–5 μm sections for pathological evaluation and 10 μm sections for FTIR analysis, generating a total of 690 sections that were deparaffinized and placed in a vacuum to remove remaining water before acquiring FTIR spectra [34]. Baseline correction of the re-processed data was performed using the AirPLS algorithm [16], followed by normalization using the SNV algorithm [17]. Figures showing FTIR-spectra were generated using MATLAB 2021a.

A convolutional neural network (CNN) based on LeNet architecture was developed in Python 3.10 to classify tissue samples. The network consisted of two convolutional layers (conv1: 16 filters, conv2: 32 filters), MaxPooling layers, and three fully connected layers. For CNN training and evaluation, the spectra obtained from patients were subsequently randomly divided into training and test sets in an approximate ratio of 70:30. In that process, all spectra of one patient were placed in the training set, and the other test set. Specifically, division was performed per patient and 30% of patients were randomly selected, whereby all spectra acquired from their samples were assigned to the test set, while the spectra from the remaining patients were assigned to the training set, preventing information leakage between partitions.

Data were standardized using StandardScaler. StandardScaler was fit on the training set and applied to the test set to prevent information leakage. The CNN was trained with the Adam optimizer (learning rate 0.001) using cross-entropy loss and a mini-batch size of 32 for a fixed schedule of 50 epochs; no early stopping or validation-based stopping criterion was applied. No class weighting, resampling, or probability calibration was used, as the four diagnostic classes were approximately balanced at the patient level (with normal pleura as the smaller group). The complete training script, including all hyperparameters and confusion-matrix generation, is provided as Supplementary File S9.

### 4.2. Sample Preparation and Isolation of N-Glycans

For the glycomic validation, a separate sample set was used, comprising archival FFPE tissue blocks obtained from the same archives of the Department of Pathology (University of Zagreb, School of Medicine). A total of 30 samples (from 30 individuals; 93% male, median age of 65.5 years, age range: 50–77 years) of normal pleura and 30 samples (from 22 patients; 90% male, median age of 63 years, age range: 36–82 years) of MPM were analyzed. This set included 10 MPM patients and 2 normal-pleura individuals whose FFPE blocks had also been analyzed by FTIR in Sadiku Zehri et al. [34], enabling direct molecular cross-verification of the FTIR findings for these individuals while the remaining patients were newly included for this glycomic analysis. Detailed per-sample age and sex information of all patients and controls is provided in Supplementary File S10.

Obtained FFPE blocks were sectioned at 5 μm thickness and mounted on glass slides. Sections were deparaffinized and rehydrated via a series of washes: 2 × 10 min in xylene, 2 × 5 min in 100% ethanol, 2 × 5 min in 96% ethanol, 2 × 5 min in 70% ethanol, and 2 × 5 min in water. Sections were then scraped off the slides with a clean scalpel and the shredded tissue pieces were transferred to 1.5 mL microfuge tubes containing Radioimmunoprecipitation Assay (RIPA) lysis buffer containing 1X cOmplete™ Mini EDTA-free Protease Inhibitor Cocktail (Roche 11836170001). The composition of the lysis buffer was as follows: 25 mM Tris-HCl pH 7.6, 150 mM NaCl, 1% Igepal-CA630, 1% sodium deoxycholate, 0.1% sodium dodecyl sulfate. Six sections from the same block were combined to form a single sample from each patient. The tubes containing the tissue pieces were vortexed and a 50 µL aliquot was taken for deglycosylation. The reducing agent β-mercaptoethanol was added to a final concentration of 0.6% *v*/*v* (i.e., 0.3 µL) after which the tubes were again vortexed and incubated at 60 °C for 10 min. The tubes were allowed to cool to room temperature at which point 5.5 µL of 10% Igepal-CA630 was added to each tube. After vortexing, 0.5 µL of PNGase F (5 units; Promega V483A, Madison, WI, USA) was added, the tubes were again vortexed, sealed with parafilm to prevent evaporation, and then incubated at 37 °C overnight. The following day, a further 0.5 µL of PNGase F was added to each tube, the tubes were vortexed, sealed with parafilm, and again incubated overnight at 37 °C. Released N-glycans were extracted from the reaction mixture using chloroform/methanol extraction as described previously [65]. Briefly, 143.5 µL of water was added to bring the total volume to 200 µL followed by 600 µL of methanol and 150 µL of chloroform. The tubes were vigorously vortexed and a further 450 µL of water was added. The tubes were again vigorously vortexed and centrifuged at maximum velocity on a benchtop centrifuge for 2 min. The aqueous phase containing the free N-glycans was collected and transferred to a clean microfuge tube. The samples were dried in a vacuum concentrator and resuspended in 50 µL of water for labeling.

### 4.3. Fluorescent Labeling of N-Glycans and Solid Phase Extraction (SPE)

Released N-glycans were labeled with procainamide (ProA) via reductive amination using a two-step protocol. First, 25 µL of 0.28 M ProA in 30% *v*/*v* acetic acid (CH_3_COOH) in dimethylsulfoxide (DMSO) was added to the extracted N-glycans after which the tubes were vortexed and incubated at 65 °C for 1 h. In the second step, 25 µL of 0.84 M 2-methylpyridine borane complex (Sigma-Aldrich 654213, Saint Louis, MO, USA) in 30% *v*/*v* CH_3_COOH in DMSO was added after which the tubes were vortexed and incubated for a further 1.5 h at 65 °C. Excess dye and other reagents were removed using hydrophilic interaction liquid chromatography (HILIC) SPE on a 0.2 μm AcroPrep™ Advance water wettable polytetrafluoroethylene (wwPTFE) 96-well membrane filter plate (Pall Corporation, Port Washington, NY, USA) with 1 mL well volume according to the following protocol. The plates were placed on a vacuum manifold and the suction pressure adjusted to just below 6750 Pa. The wells were washed by vacuum suction first with 200 μL 70% ethanol, then 200 μL water, then 200 μL of ice cold 96% acetonitrile (ACN) to condition the wells. The labeling reaction mixtures were mixed with 700 μL ice cold 100% ACN, loaded onto the wells, and allowed to incubate for 2 min before being vacuumed to waste. Four washes with 200 μL of ice cold 96% ACN on the vacuum manifold were followed by a final wash with ice cold 96% ACN by centrifugation in a benchtop centrifuge for 5 min at 1000 revolutions per minute (rpm). The plates were placed onto a clean collection plate, 90 μL of water was added to each well, and the plates were incubated for 15 min at room temperature with shaking. The labeled N-glycans were eluted into the collection plates by centrifugation in a benchtop centrifuge for 5 min at 1000 rpm. The elution step was repeated with another 90 μL of water for a final elution volume of 180 μL. Eluted N-glycans were stored at −20 °C until use.

### 4.4. Linkage-Specific Derivatization of Sialic Acids

To allow for differentiation between α(2-3)- and α(2-6)-linked Neu5Ac and to increase the stability of the modified sialic acids during UPLC, sialic acids were chemically modified using a two-step derivatization protocol. In the first step, α(2,6)-linked Neu5Ac residues are ethyl esterified (EE) at the carboxyl group, while α(2-3)-linked Neu5Ac residues form an internal lactone [66]. In the second step, the labile lactones are converted to more stable methylamides (MA) [67]. A fraction of the released and ProA-labeled N-glycans were dried using a vacuum concentrator and resuspended in 2 µL of water. Derivatization reagent containing 250 mM 1-ethyl-3-(3-dimethylaminopropyl)carbodiimide hydrochloride (EDC; Sigma-Aldrich 03450-5G) and 250 mM hydroxybenzotriazole monohydrate (HOBt; Sigma-Aldrich 54802-10G-F) in ethanol was prepared fresh and 20 µL was added to each sample. Samples were vortexed and incubated for 1 h at 37 °C after which 4 µL of methylamine (CH_3_NH_2_) solution (40% *w*/*v* in water; Sigma-Aldrich 426466-1L) was added to each sample (final CH_3_NH_2_ concentration of 6.2%). Samples were vortexed and incubated for a further 2 h at 37 °C to convert lactones to methylamides. Samples were then briefly dried in a vacuum concentrator for 30 min to evaporate the ethanol and were resuspended in 50 µL of water to prepare for clean up using porous graphitized carbon (PGC) SPE. Theoretically, this chemistry should result in mass shifts of +13.04 Da and +28.03 for MA and EE Neu5Ac, respectively, enabling the distinction of α(2-3)-linked Neu5Ac (304.14 Da) and α(2-6)-linked Neu5Ac (319.13 Da). While the expected mass shift was observed for MA Neu5Ac, for unknown reasons the mass shift for EE Neu5Ac turned out to be +169.21 Da, resulting in a mass of 460.31 Da for α(2-6)-linked Neu5Ac, which was verified on control samples of human plasma N-glycans. Although the exact elemental composition of this mismodification could not be determined, it was uniformly and selectively present on α(2-6)-linked Neu5Ac and thus still allowed for distinction between α(2-3)- and α(2-6)-linked Neu5Ac.

### 4.5. Purification of N-Glycans Using PGC SPE

To remove derivatization reagents and purify N-glycans for UPLC analysis, PGC SPE was performed using mini PGC columns made using ZipTip^®^ Pipette Tips (C18 resin, bed volume 0.6 μL, tip volume 10 μL; Merck Millipore ZTC18S096, Darmstadt, Germany). The PGC matrix was extracted from Extract-Clean™ SPE Carbo 300 mg/8 mL Cartridges (S*Pure 5122418, Vineland, NJ, USA) and resuspended in methanol at 50 mg/mL to form a PGC/methanol slurry. Then, 50 µL of the slurry (equivalent to 2.5 mg PGC) was loaded into empty ZipTip^®^ Pipette Tips situated within plastic adapters that enabled them to be placed into 2 mL microfuge tubes. All centrifugation steps were performed at 2000× *g* for 30 s. The ZipTip^®^ Pipette Tips were centrifuged to pack the columns and remove any fines. Next, the columns were washed twice with 60 μL ACN and twice with 60 μL water by centrifugation. To bind N-glycans to the PGC solid phase, the N-glycan samples were loaded on the columns and centrifuged after which the columns were washed with 60 μL water by centrifugation. At this point, the adapters containing the columns were transferred to clean 1.5 mL microfuge tubes. Bound N-glycans were eluted using 40 μL of 73% (*vol*/*vol*) ACN/27% 100 mM ammonium formate pH = 4.4. Eluted N-glycans were dried using a vacuum concentrator and resuspended in 16.8 µL of water. The N-glycan samples were brought to the starting conditions for UPLC analysis by the addition of 43.2 µL of ACN to achieve a final volume of 60 µL and a concentration of 72% ACN.

### 4.6. UPLC Analysis of N-Glycans

Released N-glycans were analyzed by hydrophilic interaction liquid chromatography (HILIC) on an ACQUITY UPLC system (Waters Corporation; Milford, MA, USA) consisting of a quaternary solvent manager, sample manager and a fluorescence detector set with the excitation and emission wavelengths of 310 and 370 nm, respectively. The instrument was under the control of Empower™ software (version 3.6.1; Waters Corporation). N-glycan separation was achieved through an ACQUITY Premier Ethylene Bridged Hybrid (BEH) Amide column (2.1 mm × 150 mm, 1.7 μm particles, 130 Å pore diameter; Waters Corporation 186009524), using 100 mM ammonium formate pH 4.4 as Solvent A and 100% MS grade ACN as Solvent B. N-glycans were prepared in 72% ACN and 50 μL of the mixture was injected into the column. Analytes were separated using a two-step linear gradient with a flowrate of 0.561 mL/min: 27.8–32.5% Solvent A in the first 48.53 min and 32.5–38.7% Solvent A for the next 45 min. Starting conditions (27.8%A/72.2% B) were held for 1.47 min to allow free ProA to elute from the column prior to the start of the gradient. Samples were maintained at 10 °C before injection, and the column temperature was 45 °C. The column was washed with 70%A/30%B for 4 min at 0.25 mL/min between injections to clean the column and prevent carryover. The 60 samples were analyzed across 15 UPLC runs. Quality control (QC) of each chromatographic run was carried out using water as a blank (negative control) and positive control samples that were run in replicate to assess instrument integrity. In this case, a pooled mixture of ProA-labeled N-glycans derived from human adipose tissue was used as a positive control and an aliquot was run within each UPLC batch. An aliquot of ProA-labeled glucose oligomers derived from partially hydrolyzed dextran was also run as an external standard within each UPLC run to enable the assignment of glucose unit (GU) values to each chromatographic segment of the pleura N-glycoprofile based on its retention time relative to the glucose ladder as previously described [68]. 10 samples failed QC (4 samples of normal pleura, and 6 samples of MPM) which resulted in their exclusion from the final data analysis, while 1 sample of normal pleura (NP24) had an insufficient amount of material for UPLC analysis. Detailed per-sample and per-patient UPLC chromatogram is provided in Supplementary File S6. Samples that failed QC are labeled with *. Owing to the complexity of the chromatograms, integration of chromatograms was performed manually using the Empower™ software to reproduce the same integration pattern for all samples. All chromatograms were integrated the same manner, dividing the chromatographic profile into 49 segments (or peaks), and the proportion of N-glycans inside each segment was expressed as a percentage of the total integrated area (total area normalization).

### 4.7. Tandem MS Analysis of ProA-Labeled N-Glycans

LC-MS analysis of released N-glycans was performed as described previously [69], with minor modifications. Briefly, biological replicates belonging to the same experimental group were pooled to create a single representative sample for each group (Control and MPM) that was then used for qualitative structural analysis by UPLC coupled to a Bruker Compact QqTOF mass spectrometer under the control of the HyStar software, version 3.2 (Bruker, Bremen, Germany). In this setup, electrospray ionization (ESI) was combined with a quadrupole-time-of-flight (QqTOF) mass analyzer to achieve HILIC-UPLC-ESI-QqTOF MS/MS profiling of ProA-labeled released N-glycans. The chromatographic gradient was the same as described above, and the mass spectrometer was set up as follows: positive ion mode, ionBooster source, capillary voltage 3.6 kV, dry gas temperature 180 °C and flow 4 L/min. The mass-to-charge ratio (*m*/*z*) range over which mass spectra were recorded was from 45 to 3000 *m*/*z* and spectra were acquired at a frequency of 0.5 Hz. Tandem MS experiments were performed in the data-dependent acquisition mode with the three precursors of highest intensity being selected to undergo collision-induced dissociation (CID) fragmentation. The *m*/*z* range for MS/MS spectra was also 45–3000 *m*/*z*. Spectra were processed in Bruker Compass Data Analysis 4.4. The acquired data were analyzed manually, wherein N-glycan compositions were proposed via searching GlycoMod (http://web.expasy.org/glycomod, accessed on 22–30 September and 1–4 October 2025) [70] for monoisotopic masses of the ions detected in the base peak chromatogram, with mass tolerance of ±0.5 Daltons. Glycan compositions and structural features were manually confirmed using tandem MS fragmentation data, where possible, which were searched for diagnostic product ions. The proposed structures were then depicted using GlycoWorkbench 2.1 stable build 146 [71] according to the symbolic representation of glycans as proposed by the Consortium for Functional Glycomics [72].

### 4.8. UPLC Data Curation and Statistical Analysis

To ensure the highest quality dataset, UPLC data underwent QC to exclude substandard samples (e.g., those with inadequate signal intensity or unsatisfactory chromatographic separation). The number of samples remaining following QC were: normal pleura n = 25, MPM pleura n = 24. Raw UPLC data are available in Supplement File S5. Due to the right skewness of their distributions, all N-glycan peak abundance data were log2-transformed prior to statistical analyses to enable the use of parametric tests. All statistical tests were performed in GraphPad prism (version 10.4.2). Differences in the abundance of individual N-glycan peaks between the groups were tested using multiple unpaired *t* tests with Welch correction with correction for multiple comparisons using a two-stage step-up False Discovery Rate (FDR) method. The FDR was set to 1%. PLS-DA analysis was performed and figures created using MATLAB 2021a with PLS Toolbox 9.0. Input data for the PLS-DA were relative peak abundance for all N-glycan peaks in the N-glycoprofile which were then pre-processed with Mean Center method. Code, confusion matrix and model for PLS-DA can be found in Supplement File S11. Note that only N-glycan peaks were included in all the performed analysis—N-glycan peaks overlapping with free oligosaccharide peaks were excluded (Figure 3).

Generative artificial intelligence (GenAI) has been used to translate parts of the text originally written in Croatian, edit and format text. The authors have reviewed and edited the output and take full responsibility for the content of this publication.

## 5. Conclusions

FTIR spectroscopy combined with machine learning algorithms enables rapid differentiation of pleural pathological conditions with high accuracy. The spectral differences are primarily located in the glycan absorption region and correlate with extensive N-glycosylation changes in comparison to normal pleura. In combination with UPLC and MS, our experiments identified a distinct glycan fingerprint of MPM, providing biological insight into the molecular basis of spectral biomarkers. This approach identified several high-mannose N-glycans, namely H5N2 and H7N2 as well as isomer switching and phosphorylation changes in H6N2 and H8N2 that offer significant biomarker potential for differential MPM diagnosis and should be further examined in larger studies.

## Figures and Tables

**Table 1:** Spectral contribution analysis with 50 cm^−1^ wavelength steps. All areas of the spectra with accuracy higher than 0.7 are listed in the table and types of biological molecules typically associated with the given range.

Spectrum Ranges (50 cm^−1^) Associated with Biological Molecules and Type of Molecules Primarily Associated with Given Range	Wavelength (cm^−1^)	Accuracy	Precision	Sensitivity/Recall	Specificity
Carbohydrates/glycans	1000–1050	0.7895	0.7907	0.7895	0.8006
	1050–1100	0.7368	0.7545	0.7368	0.7579
	1100–1150	0.8026	0.8129	0.8026	0.8184
	1150–1200	0.9737	0.9750	0.9737	0.9749
Nucleic acids	1200–1250	0.8684	0.8687	0.8684	0.8738
	1250–1300	0.8816	0.8847	0.8816	0.8911
	1300–1350	0.7895	0.8226	0.7895	0.7945
Nucleic acids/proteins	1350–1400	0.7895	0.7884	0.7895	0.7945
	1400–1450	0.7500	0.7553	0.7500	0.7666
Proteins	1450–1500	0.8684	0.8727	0.8684	0.8855
	1650–1700	0.8684	0.8745	0.8684	0.8826
	1700–1750	0.8026	0.8191	0.8026	0.8278
Lipids	2850–2900	0.7869	0.7907	0.7763	0.7938
	2900–2950	0.7763	0.7792	0.7763	0.7894
	2950–3000	0.8289	0.8433	0.8289	0.8525
	3000–3050	0.8026	0.8060	0.8026	0.8059
	3050–3100	0.7368	0.7377	0.7368	0.7472

**Table 2:** Reported relative changes in high-mannose glycan abundance in different lung pathologies. Expression levels of H5N2, H7N2, H6N2 and H8N2 N-glycans in different lung pathologies compared to the controls. Symbols: - indicates no significant change, green arrows indicate decreased expression and red arrows indicate increased expression. Isomer switching and/or phosphorylation changes are indicated as I/P.

Source	Ruhaak et al. 2015 [47]	Lattova et al. 2020 [48]	Lattova et al. 2025 [46]	Lattova et al. 2025 [46]	Current Study (Kavur et al.)
Lung Pathology	Lung Adenocarcinoma	Lung Adenocarrcinoma	Multiple LC Types (LAC, SqCC, SCLC, Sec-LAc)	Inflammation	MPM
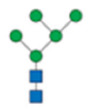 H5N2	-	-	-	-	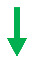
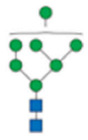 H7N2	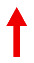	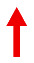	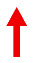	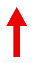	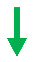
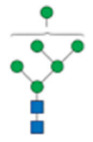 H6N2	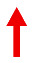	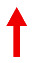	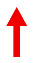	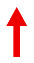	I/P
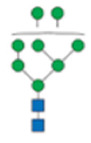 H8N2	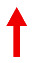	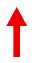	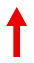	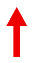	I/P

## Data Availability

The original contributions presented in this study are included in the article/Supplementary Materials. Further inquiries can be directed to the corresponding authors.

## Supplementary Materials

The following supporting information can be downloaded at: https://www.mdpi.com/article/10.3390/ijms27146134/s1.

## Abbreviations

The following abbreviations are used in this manuscript:

MPM	Malignant Pleural Mesothelioma
FTIR	Fourier-transform Infrared Spectroscopy
CNN	Convolutional Neural Network
UPLC	Ultra-high-Performance Liquid Chromatography
MS	Mass spectrometry
FF	Formalin-Fixed
FFPE	Formalin-Fixed Paraffin-Embedded

## References

[1] Scherpereel A., Opitz I., Berghmans T., Psallidas I., Glatzer M., Rigau D., Astoul P., Bölükbas S., Boyd J., Coolen J. (2020). ERS/ESTS/EACTS/ESTRO Guidelines for the Management of Malignant Pleural Mesothelioma. Eur. Respir. J..

[2] Carbone M., Ly B.H., Dodson R.F., Pagano I., Morris P.T., Dogan U.A., Gazdar A.F., Pass H.I., Yang H. (2012). Malignant Mesothelioma: Facts, Myths, and Hypotheses. J. Cell. Physiol..

[3] Hemminki K., Försti A., Chen T., Hemminki A. (2021). Incidence, Mortality and Survival in Malignant Pleural Mesothelioma before and after Asbestos in Denmark, Finland, Norway and Sweden. BMC Cancer.

[4] Huang J., Chan S.C., Pang W.S., Chow S.H., Lok V., Zhang L., Lin X., Lucero-Prisno D.E., Xu W., Zheng Z.-J. (2023). Global Incidence, Risk Factors, and Temporal Trends of Mesothelioma: A Population-Based Study. J. Thorac. Oncol..

[5] Zhai Y., Hui Z., Chen W., Ying J., Li J., Gao S. (2022). The Epidemic of Malignant Mesothelioma in China: A Prediction of Incidence during 2016–2030. Transl. Lung Cancer Res..

[6] Brims F. (2021). Epidemiology and Clinical Aspects of Malignant Pleural Mesothelioma. Cancers.

[7] Husain A.N., Colby T., Ordonez N., Krausz T., Attanoos R., Beasley M.B., Borczuk A.C., Butnor K., Cagle P.T., Chirieac L.R. (2013). Guidelines for Pathologic Diagnosis of Malignant Mesothelioma: 2012 Update of the Consensus Statement from the International Mesothelioma Interest Group. Arch. Pathol. Lab. Med..

[8] Husain A.N., Colby T.V., Ordóñez N.G., Allen T.C., Attanoos R.L., Beasley M.B., Butnor K.J., Chirieac L.R., Churg A.M., Dacic S. (2017). Guidelines for Pathologic Diagnosis of Malignant Mesothelioma: 2017 Update of the Consensus Statement from the International Mesothelioma Interest Group. Arch. Pathol. Lab. Med..

[9] Cigognetti M., Lonardi S., Fisogni S., Balzarini P., Pellegrini V., Tironi A., Bercich L., Bugatti M., Rossi G., Murer B. (2015). BAP1 (BRCA1-Associated Protein 1) Is a Highly Specific Marker for Differentiating Mesothelioma from Reactive Mesothelial Proliferations. Mod. Pathol..

[10] Woolhouse I., Bishop L., Darlison L., Fonseka D.D., Edey A., Edwards J., Faivre-Finn C., Fennell D.A., Holmes S., Kerr K.M. (2018). British Thoracic Society Guideline for the Investigation and Management of Malignant Pleural Mesothelioma. Thorax.

[11] Bibby A.C., Tsim S., Kanellakis N., Ball H., Talbot D.C., Blyth K.G., Maskell N.A., Psallidas I. (2016). Malignant Pleural Mesothelioma: An Update on Investigation, Diagnosis and Treatment. Eur. Respir. Rev..

[12] Ledda C., Senia P., Rapisarda V. (2018). Biomarkers for Early Diagnosis and Prognosis of Malignant Pleural Mesothelioma: The Quest Goes On. Cancers.

[13] Wang Y., Liu Y., Liu S., Cheng L., Liu X. (2024). Recent Advances in N-Glycan Biomarker Discovery among Human Diseases. Acta Biochim. Biophys. Sin..

[14] Ruhaak L.R., Miyamoto S., Lebrilla C.B. (2013). Developments in the Identification of Glycan Biomarkers for the Detection of Cancer. Mol. Cell. Proteom..

[15] Pinho S.S., Reis C.A. (2015). Glycosylation in Cancer: Mechanisms and Clinical Implications. Nat. Rev. Cancer.

[16] Paton B., Herrero P., Peraire J., del Pino A., Chafino S., Martinez-Picado J., Gómez-Bertomeu F., Rull A., Canela N., Suárez M. (2023). Fucosylated N-Glycans as Early Biomarkers of COVID-19 Severity. Front. Immunol..

[17] Balbisi M., Sugár S., Turiák L. (2026). Protein Glycosylation in Lung Cancer from a Mass Spectrometry Perspective. Mass Spectrom. Rev..

[18] Alvarez M.R., Zhou Q., Tena J., Barboza M., Wong M., Xie Y., Lebrilla C.B., Cabanatan M., Barzaga M.T., Tan-Liu N. (2023). Glycomic, Glycoproteomic, and Proteomic Profiling of Philippine Lung Cancer and Peritumoral Tissues: Case Series Study of Patients Stages I–III. Cancers.

[19] Shkunnikova S., Mijakovac A., Sironic L., Hanic M., Lauc G., Kavur M.M. (2023). IgG Glycans in Health and Disease: Prediction, Intervention, Prognosis, and Therapy. Biotechnol. Adv..

[20] Fujihira H., Takakura D., Matsuda A., Abe M., Miyazaki M., Nakagawa T., Kajino K., Denda-Nagai K., Noji M., Hino O. (2021). Bisecting-GlcNAc on Asn388 Is Characteristic to ERC/Mesothelin Expressed on Epithelioid Mesothelioma Cells. J. Biochem..

[21] Nakashima K., Sakai Y., Hoshino H., Umeda Y., Kawashima H., Sekido Y., Ishizuka T., Kobayashi M. (2022). Sulfated Glycans Recognized by S1 Monoclonal Antibody Can Serve as a Diagnostic Marker for Malignant Pleural Mesothelioma. Lung.

[22] Cortes-Dericks L., Schmid R.A. (2017). CD44 and Its Ligand Hyaluronan as Potential Biomarkers in Malignant Pleural Mesothelioma: Evidence and Perspectives. Respir. Res..

[23] Creaney J., Robinson B.W. (2009). Serum and Pleural Fluid Biomarkers for Mesothelioma. Curr. Opin. Pulm. Med..

[24] Chen Z., Gaudino G., Pass H.I., Carbone M., Yang H. (2017). Diagnostic and Prognostic Biomarkers for Malignant Mesothelioma: An Update. Transl. Lung Cancer Res..

[25] Karunakaran K.B., Yanamala N., Boyce G., Becich M.J., Ganapathiraju M.K. (2021). Malignant Pleural Mesothelioma Interactome with 364 Novel Protein-Protein Interactions. Cancers.

[26] Lucà S., Pignata G., Cioce A., Salzillo C., Cecio R.D., Ferrara G., Corte C.M.D., Morgillo F., Fiorelli A., Montella M. (2025). Diagnostic Challenges in the Pathological Approach to Pleural Mesothelioma. Cancers.

[27] Baker M.J., Hussain S.R., Lovergne L., Untereiner V., Hughes C., Lukaszewski R.A., Thiéfin G., Sockalingum G.D. (2015). Developing and Understanding Biofluid Vibrational Spectroscopy: A Critical Review. Chem. Soc. Rev..

[28] Su K.-Y., Lee W.-L. (2020). Fourier Transform Infrared Spectroscopy as a Cancer Screening and Diagnostic Tool: A Review and Prospects. Cancers.

[29] Simonova D., Karamancheva I. (2013). Application of Fourier Transform Infrared Spectroscopy for Tumor Diagnosis. Biotechnol. Biotechnol. Equip..

[30] Baker M.J., Gazi E., Brown M.D., Shanks J.H., Gardner P., Clarke N.W. (2008). FTIR-Based Spectroscopic Analysis in the Identification of Clinically Aggressive Prostate Cancer. Br. J. Cancer.

[31] Lima K.M.G., Gajjar K.B., Martin-Hirsch P.L., Martin F.L. (2015). Segregation of Ovarian Cancer Stage Exploiting Spectral Biomarkers Derived from Blood Plasma or Serum Analysis: ATR-FTIR Spectroscopy Coupled with Variable Selection Methods. Biotechnol. Prog..

[32] Zhang X., Thiéfin G., Gobinet C., Untereiner V., Taleb I., Bernard-Chabert B., Heurgué A., Truntzer C., Ducoroy P., Hillon P. (2013). Profiling Serologic Biomarkers in Cirrhotic Patients via High-Throughput Fourier Transform Infrared Spectroscopy: Toward a New Diagnostic Tool of Hepatocellular Carcinoma. Transl. Res..

[33] Yonar D., Severcan M., Gurbanov R., Sandal A., Yilmaz U., Emri S., Severcan F. (2022). Rapid Diagnosis of Malignant Pleural Mesothelioma and Its Discrimination from Lung Cancer and Benign Exudative Effusions Using Blood Serum. Biochim. Biophys. Acta (BBA)-Mol. Basis Dis..

[34] Sadiku-Zehri F., Gamulin O., Škrabić M., Qerimi-Krasniqi A., Sedlić F., Šepac A., Brčić L., Vuletić L.B., Seiwerth S. (2020). Differentiating Between Malignant Mesothelioma and Other Pleural Lesions Using Fourier Transform Infrared Spectroscopy. Appl. Spectrosc..

[35] Derenne A., Derfoufi K.-M., Cowper B., Delporte C., Goormaghtigh E. (2020). FTIR Spectroscopy as an Analytical Tool to Compare Glycosylation in Therapeutic Monoclonal Antibodies. Anal. Chim. Acta.

[36] Derenne A., Derfoufi K.-M., Cowper B., Delporte C., Butré C.I., Goormaghtigh E. (2021). Analysis of Glycoproteins by ATR-FTIR Spectroscopy: Comparative Assessment. Methods in Molecular Biology.

[37] Hamla S., Sacré P.-Y., Derenne A., Derfoufi K.-M., Cowper B., Butré C.I., Delobel A., Goormaghtigh E., Hubert P., Ziemons E. (2022). A New Alternative Tool to Analyse Glycosylation in Pharmaceutical Proteins Based on Infrared Spectroscopy Combined with Nonlinear Support Vector Regression. Analyst.

[38] Eissa T., Voronina L., Huber M., Fleischmann F., Žigman M. (2024). The Perils of Molecular Interpretations from Vibrational Spectra of Complex Samples. Angew. Chem. Int. Ed..

[39] Voronina L., Fleischmann F., Šimunović J., Ludwig C., Novokmet M., Žigman M. (2024). Probing Blood Plasma Protein Glycosylation with Infrared Spectroscopy. Anal. Chem..

[40] Abbas S., Ozek N.S., Emri S., Koksal D., Severcan M., Severcan F. (2018). Diagnosis of Malignant Pleural Mesothelioma from Pleural Fluid by Fourier Transform-Infrared Spectroscopy Coupled with Chemometrics. J. Biomed. Opt..

[41] Matsuura R., Kaji H., Tomioka A., Sato T., Narimatsu H., Moriwaki Y., Misawa H., Imai K., Tsuji S. (2018). Identification of Mesothelioma-Specific Sialylated Epitope Recognized with Monoclonal Antibody SKM9-2 in a Mucin-like Membrane Protein HEG1. Sci. Rep..

[42] Vajaria B.N., Patel P.S. (2017). Glycosylation: A Hallmark of Cancer?. Glycoconj. J..

[43] Conroy L.R., Chang J.E., Sun Q., Clarke H.A., Buoncristiani M.D., Young L.E.A., McDonald R.J., Liu J., Gentry M.S., Allison D.B. (2022). High-Dimensionality Reduction Clustering of Complex Carbohydrates to Study Lung Cancer Metabolic Heterogeneity. Adv. Cancer Res..

[44] Satomaa T., Heiskanen A., Leonardsson I., Ångström J., Olonen A., Blomqvist M., Salovuori N., Haglund C., Teneberg S., Natunen J. (2009). Analysis of the Human Cancer Glycome Identifies a Novel Group of Tumor-Associated N-Acetylglucosamine Glycan Antigens. Cancer Res..

[45] Wang X., Deng Z., Huang C., Zhu T., Lou J., Wang L., Li Y. (2018). Differential N-Glycan Patterns Identified in Lung Adenocarcinoma by N-Glycan Profiling of Formalin-Fixed Paraffin-Embedded (FFPE) Tissue Sections. J. Proteom..

[46] Lattova E., Skrickova J., Hausnerova J., Krystofova K., Zdrahal Z., Kren L., Popovic M. (2025). N-Glycans in Lung Tissue Specimens: A Prospective Target for Enhanced Cancer Diagnosis and Prognosis. J. Transl. Med..

[47] Ruhaak L.R., Taylor S.L., Stroble C., Nguyen U.T., Parker E.A., Song T., Lebrilla C.B., Rom W.N., Pass H., Kim K. (2015). Differential N-Glycosylation Patterns in Lung Adenocarcinoma Tissue. J. Proteome Res..

[48] Lattova E., Skřičková J., Hausnerová J., Frola L., Křen L., Ihnatová I., Zdráhal Z., Bryant J., Popovič M. (2020). N-Glycan Profiling of Lung Adenocarcinoma in Patients at Different Stages of Disease. Mod. Pathol..

[49] Donczo B., Kiraly G., Guttman A. (2019). Effect of the Elapsed Time between Sampling and Formalin Fixation on the N-glycosylation Profile of Mouse Tissue Specimens. Electrophoresis.

[50] Donczo B., Szigeti M., Ostoros G., Gacs A., Tovari J., Guttman A. (2016). N-Glycosylation Analysis of Formalin Fixed Paraffin Embedded Samples by Capillary Electrophoresis. Electrophoresis.

[51] Jia N., Byrd-Leotis L., Matsumoto Y., Gao C., Wein A.N., Lobby J.L., Kohlmeier J.E., Steinhauer D.A., Cummings R.D. (2020). The Human Lung Glycome Reveals Novel Glycan Ligands for Influenza A Virus. Sci. Rep..

[52] Donovan M., Duke R., Simonetti L., Cavusoglu N., Rudd P.M., Bernard D. (2023). N-Glycans Are Stratum Corneum Biomarkers of Aging Skin. J. Investig. Dermatol..

[53] Oinam L., Changarathil G., Raja E., Ngo Y.X., Tateno H., Sada A., Yanagisawa H. (2020). Glycome Profiling by Lectin Microarray Reveals Dynamic Glycan Alterations During Epidermal Stem Cell Aging. Aging Cell.

[54] Veličković D., Purkerson J., Bhotika H., Huyck H., Clair G., Pryhuber G.S., Anderton C. (2025). Integrating N -Glycan and CODEX Imaging Reveal Cell-Specific Protein Glycosylation in Healthy Human Lung. Mol. Omics.

[55] Baker M.J., Trevisan J., Bassan P., Bhargava R., Butler H.J., Dorling K.M., Fielden P.R., Fogarty S.W., Fullwood N.J., Heys K.A. (2014). Using Fourier Transform IR Spectroscopy to Analyze Biological Materials. Nat. Protoc..

[56] Delrue C., Bruyne S.D., Speeckaert M.M. (2025). The Promise of Infrared Spectroscopy in Liquid Biopsies for Solid Cancer Detection. Diagnostics.

[57] Gao C., Stavenhagen K., Eckmair B., McKitrick T.R., Mehta A.Y., Matsumoto Y., McQuillan A.M., Hanes M.S., Eris D., Baker K.J. (2021). Differential Recognition of Oligomannose Isomers by Glycan-Binding Proteins Involved in Innate and Adaptive Immunity. Sci. Adv..

[58] Pocheć E., Bocian K., Ząbczyńska M., Korczak-Kowalska G., Lityńska A. (2015). Immunosuppressive Drugs Affect High-Mannose/Hybrid N-Glycans on Human Allostimulated Leukocytes. Anal. Cell. Pathol..

[59] Seo J., Oh D.-B. (2022). Mannose-6-Phosphate Glycan for Lysosomal Targeting: Various Applications from Enzyme Replacement Therapy to Lysosome-Targeting Chimeras. Anim. Cells Syst..

[60] Pohl S., Marschner K., Storch S., Braulke T. (2009). Glycosylation- and Phosphorylation-Dependent Intracellular Transport of Lysosomal Hydrolases. Biol. Chem..

[61] Hosako M., Muto T., Nakamura Y., Tsuta K., Tochigi N., Tsuda H., Asamura H., Tomonaga T., Kawai A., Kondo T. (2012). Proteomic Study of Malignant Pleural Mesothelioma by Laser Microdissection and Two-Dimensional Difference Gel Electrophoresis Identified Cathepsin D as a Novel Candidate for a Differential Diagnosis Biomarker. J. Proteom..

[62] Choi H., Ko Y., Lee C.Y. (2020). Pro-Cathepsin D as a Diagnostic Marker in Differentiating Malignant Pleural Effusion from Benign Pleural Effusion: A Retrospective Cohort Study. BMC Cancer.

[63] Rochefort H., Liaudet-Coopman E. (1999). Cathepsin D in Cancer Metastasis: A Protease and a Ligand. APMIS.

[64] Sato Y., Suzuki Y., Ito E., Shimazaki S., Ishida M., Yamamoto T., Yamamoto H., Toda T., Suzuki M., Suzuki A. (2006). Identification and Characterization of an Increased Glycoprotein in Aging: Age-Associated Translocation of Cathepsin D. Mech. Ageing Dev..

[65] Vacchini M., Cipolla L., Gornik O., Lauc G., Klarić T. (2020). A Precise and Versatile Platform for Rapid Glycosylation Analysis of Brain Tissue. Anal. Methods.

[66] Reiding K.R., Lonardi E., Ederveen A.L.H., Wuhrer M. (2015). Ethyl Esterification for MALDI-MS Analysis of Protein Glycosylation. Methods Mol. Biol..

[67] de Haan N., Yang S., Cipollo J., Wuhrer M. (2020). Glycomics Studies Using Sialic Acid Derivatization and Mass Spectrometry. Nat. Rev. Chem..

[68] Guile G.R., Rudd P.M., Wing D.R., Prime S.B., Dwek R.A. (1996). A Rapid High-Resolution High-Performance Liquid Chromatographic Method for Separating Glycan Mixtures and Analyzing Oligosaccharide Profiles. Anal. Biochem..

[69] Cindrić A., Vučković F., Murray A., Klarić T.S., Alić I., Krištić J., Nižetić D., Lauc G. (2025). Total Cell N-Glycosylation Is Altered during Differentiation of Induced Pluripotent Stem Cells to Neural Stem Cells and Is Disturbed by Trisomy 21. BBA Adv..

[70] Cooper C.A., Gasteiger E., Packer N.H. (2001). GlycoMod—A Software Tool for Determining Glycosylation Compositions from Mass Spectrometric Data. Proteomics.

[71] Ceroni A., Maass K., Geyer H., Geyer R., Dell A., Haslam S.M. (2008). GlycoWorkbench: A Tool for the Computer-Assisted Annotation of Mass Spectra of Glycans. J. Proteome Res..

[72] Varki A., Cummings R.D., Esko J.D., Freeze H.H., Stanley P., Marth J.D., Bertozzi C.R., Hart G.W., Etzler M.E. (2009). Symbol Nomenclature for Glycan Representation. Proteomics.

