# Supplementary material for: Glycan Fingerprint of Malignant Pleural Mesothelioma
Source: Int J Mol Sci. 2026 Jul 9;27(14):6134. doi: 10.3390/ijms27146134 (PMC13410575; doi:10.3390/ijms27146134)
Supplement: Supplementary file 1 [file ijms-27-06134-s001.zip › S11_File_PLSDA_CM_and_Model.pdf]

Supplementary S11

PLS-DA Confusion matrix (A) and model (B)

A) Matrix

PLSDA Classification Using Rule: Pred Most Probable

MODEL RESULTS

Confusion Matrix:

Class:	TPR	FPR	TNR	FNR	N	Err	P	F1
Class 1	1.00000	0.00000	1.00000	0.00000	25	0.00000	1.00000	1.00000
Class 2	1.00000	0.00000	1.00000	0.00000	24	0.00000	1.00000	1.00000

Matthew's Correlation Coefficient = 1.000

Confusion Table:

	Actual Class	
	Class 1	Class 2
Predicted as Class 1	25	0
Predicted as Class 2	0	24
Predicted as Unassigned	0	0

CV RESULTS

Confusion Matrix (CV):

Class:	TPR	FPR	TNR	FNR	N	Err	P	F1
Class 1	1.00000	0.04167	0.95833	0.00000	25	0.02041	0.96154	0.98039
Class 2	0.95833	0.00000	1.00000	0.04167	24	0.02041	1.00000	0.97872

Matthew's Correlation Coefficient = 0.960

Confusion Table (CV):

	Actual Class	
	Class 1	Class 2

Predicted as Class 1	25	1
Predicted as Class 2	0	23
Predicted as Unassigned	0	0

PLSDA Classification Using Rule: Pred Strict (using strictthreshold = 0.50)

MODEL RESULTS

Confusion Matrix:

Class:	TPR	FPR	TNR	FNR	N	Err	P	F1
Class 1	1.00000	0.00000	1.00000	0.00000	25	0.00000	1.00000	1.00000
Class 2	1.00000	0.00000	1.00000	0.00000	24	0.00000	1.00000	1.00000

Matthew's Correlation Coefficient = 1.000

Confusion Table:

	Actual Class	
	Class 1	Class 2
Predicted as Class 1	25	0
Predicted as Class 2	0	24
Predicted as Unassigned	0	0

CV RESULTS

Confusion Matrix (CV):

Class:	TPR	FPR	TNR	FNR	N	Err	P	F1
Class 1	1.00000	0.04167	0.95833	0.00000	25	0.02041	0.96154	0.98039
Class 2	0.95833	0.00000	1.00000	0.04167	24	0.02041	1.00000	0.97872

Matthew's Correlation Coefficient = 0.960

Confusion Table (CV):

Actual Class

	Class 1	Class 2
Predicted as Class 1	25	1
Predicted as Class 2	0	23
Predicted as Unassigned	0	0

<html><font color="#000099">See Help menu for content information

B) Model

This is a model of type: PLSDA

Developed 13-Oct-2025 19:36:26.302

Author: @DESKTOP-0SNIAD6

X-block: 49 by 49 (@DESKTOP-0SNIAD6@20251013T182946.17753225 m:20251013183032.571)

Included: [1-49] [1-49]

Preprocessing: Mean Center

Y-block: y 49 by 2 (@DESKTOP-0SNIAD6@20251013T183540.93389220 m:20251013183540.948)

Included: [1-49] [1-2]

Preprocessing: Autoscale

Num. LVs: 3

Cross validation: venetian blinds w/ 10 splits and blind thickness = 1

Statistics for each y-block column:

Modeled Class: 1 2

Sensitivity (Cal): 1.000 1.000

Specificity (Cal): 1.000 1.000

Sensitivity (CV): 0.960 0.958

Specificity (CV): 0.958 0.960

Class. Err (Cal): 0 0

Class. Err (CV): 0.0408333 0.0408333

RMSEC: 0.173074 0.173074

RMSECV: 0.239342 0.239342

Bias: -1.11022e-16 0

CV Bias: 0.00835639 -0.00835639

R^2 Cal: 0.880132 0.880132

R^2 CV: 0.773596 0.773596

Class Err. = average of false positive rate and false negative rate for class,

= 1 - (sensitivity+specificity)/2.

Percent Variance Captured by Regression Model

	-----X-Block-----		-----Y-Block-----	
Comp	This	Total	This	Total
----	-------	-------	-------	-------
1	30.78	30.78	67.35	67.35
2	20.21	50.99	12.81	80.16
3	20.57	71.56	7.85	88.01
