# Supplementary material for: Glycan Fingerprint of Malignant Pleural Mesothelioma
Source: Int J Mol Sci. 2026 Jul 9;27(14):6134. doi: 10.3390/ijms27146134 (PMC13410575; doi:10.3390/ijms27146134)

Supplementary figure

a) Confusion matrix differentiating MPM (3) from adenocarcinoma metastases (2) and b) Confusion matrix differentiating MPM (3) from Inflammation (1)

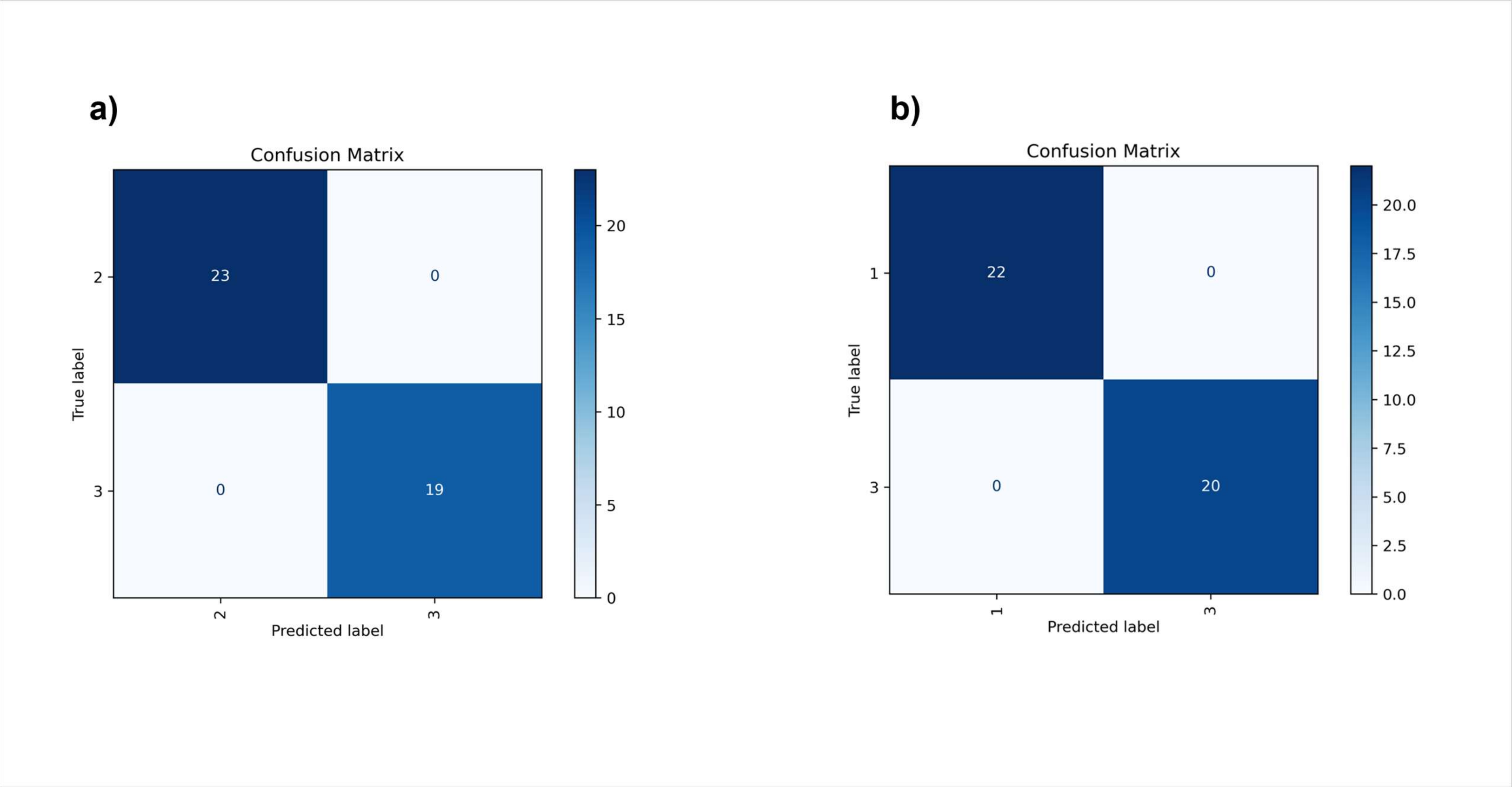

Supplement: Supplementary file 1 [file ijms-27-06134-s001.zip › S1_Confusion_Matrix.pdf]
