# Supplementary material for: Glycan Fingerprint of Malignant Pleural Mesothelioma
Source: Int J Mol Sci. 2026 Jul 9;27(14):6134. doi: 10.3390/ijms27146134 (PMC13410575; doi:10.3390/ijms27146134)

Supplementary Figure

Individual unprocessed, raw UPLC chromatograms of all samples. Samples that failed QC are labeled with *.

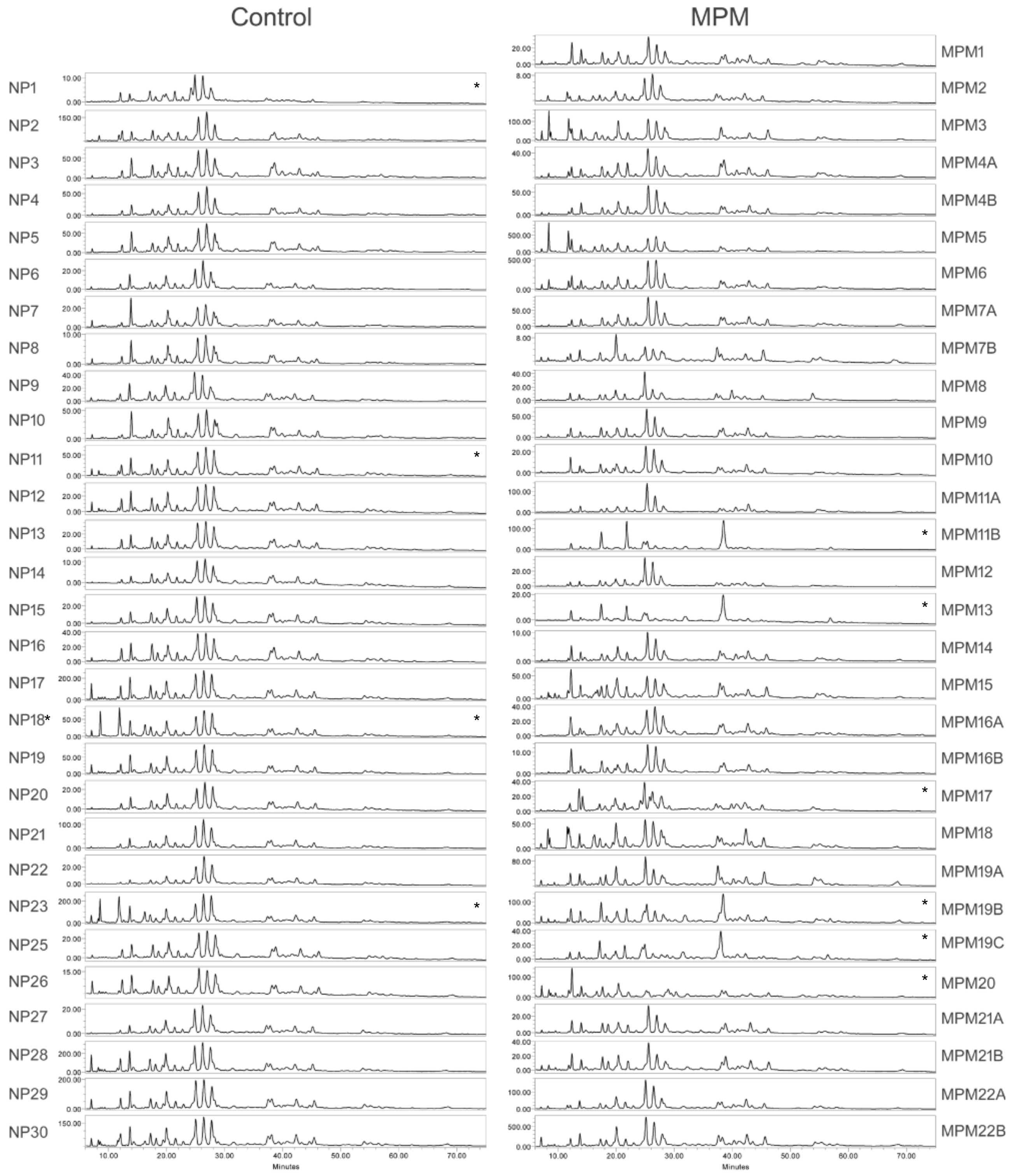

Supplement: Supplementary file 1 [file ijms-27-06134-s001.zip › S6_Figure_Raw_UPLC_Chromatograms.pdf]
