# Supplementary material for: Glycan Fingerprint of Malignant Pleural Mesothelioma
Source: Int J Mol Sci. 2026 Jul 9;27(14):6134. doi: 10.3390/ijms27146134 (PMC13410575; doi:10.3390/ijms27146134)
Supplement: Supplementary file 1 [file ijms-27-06134-s001.zip › S7_Figure_Summed_MS_Spectra.pdf]

Supplementary Figure

Summed MS spectra for N-glycan peaks whose abundance is significantly different between the Control (left) and MPM group (right). The time intervals over which the spectra were summed, as well as the scan numbers, are shown in the top right corner of the spectra. Ion intensity is shown on the y-axis, while m/z is shown on the x-axis. Major ion signals are labelled with their m/z values and charge state. Ions marked by a red diamond and red font were selected for fragmentation. The proposed structure of the dominant N-glycan is displayed in the top left corner of each spectrum. Generally, spectra from equivalent N-glycan peaks were similar between the groups. However, note that in some N-glycan peaks the identity of the dominant N-glycan varied between groups, e.g. N-glycan peaks 14, 27, and 30. Some of the ion masses (marked with an asterisk) could not be matched to biologically plausible N-glycan structures; in these cases, the next most intense ion was taken to be the dominant N-glycan.

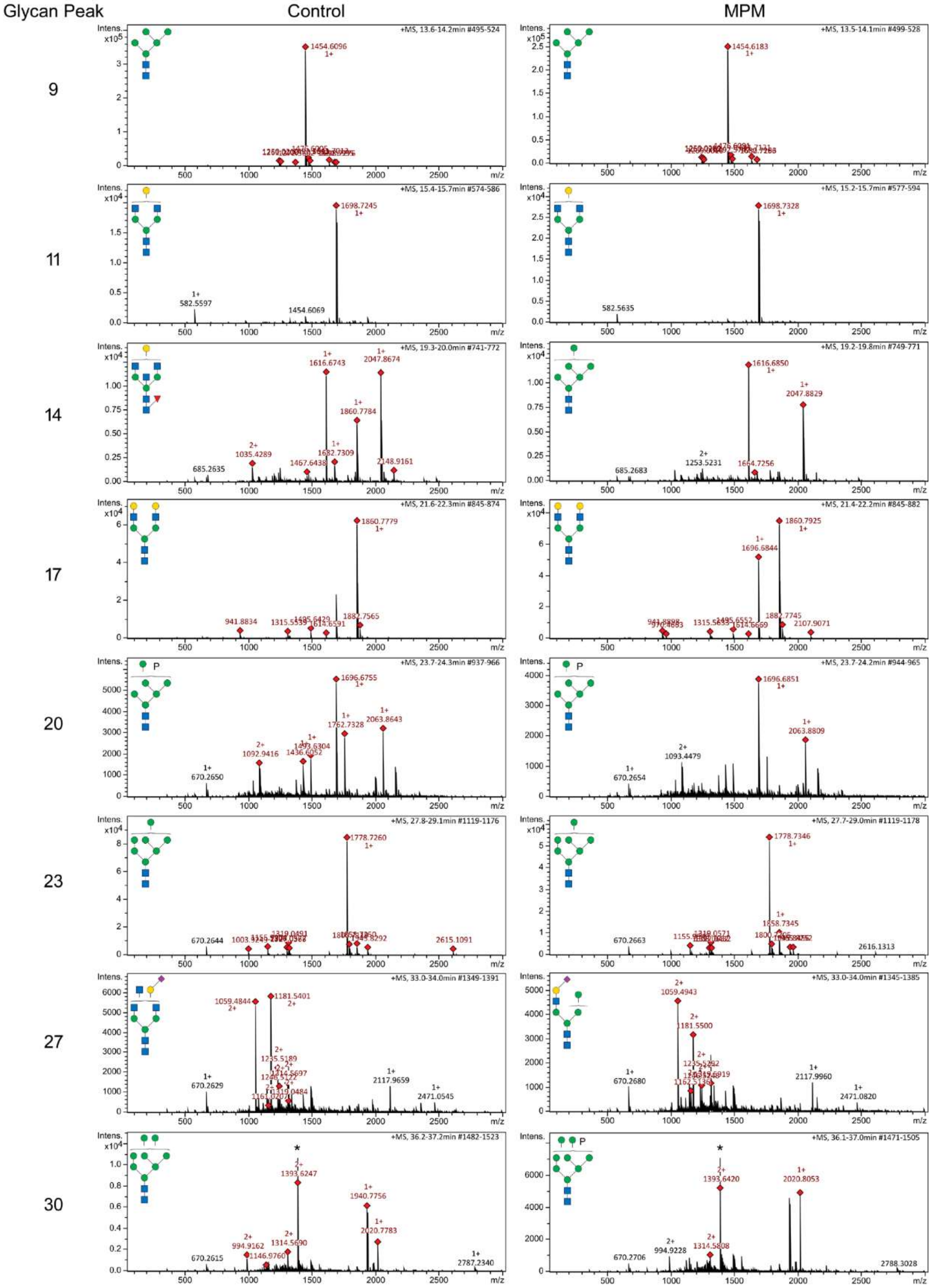
Glycan Peak
Control
MPM
9
11
14
17
20
23
27
30

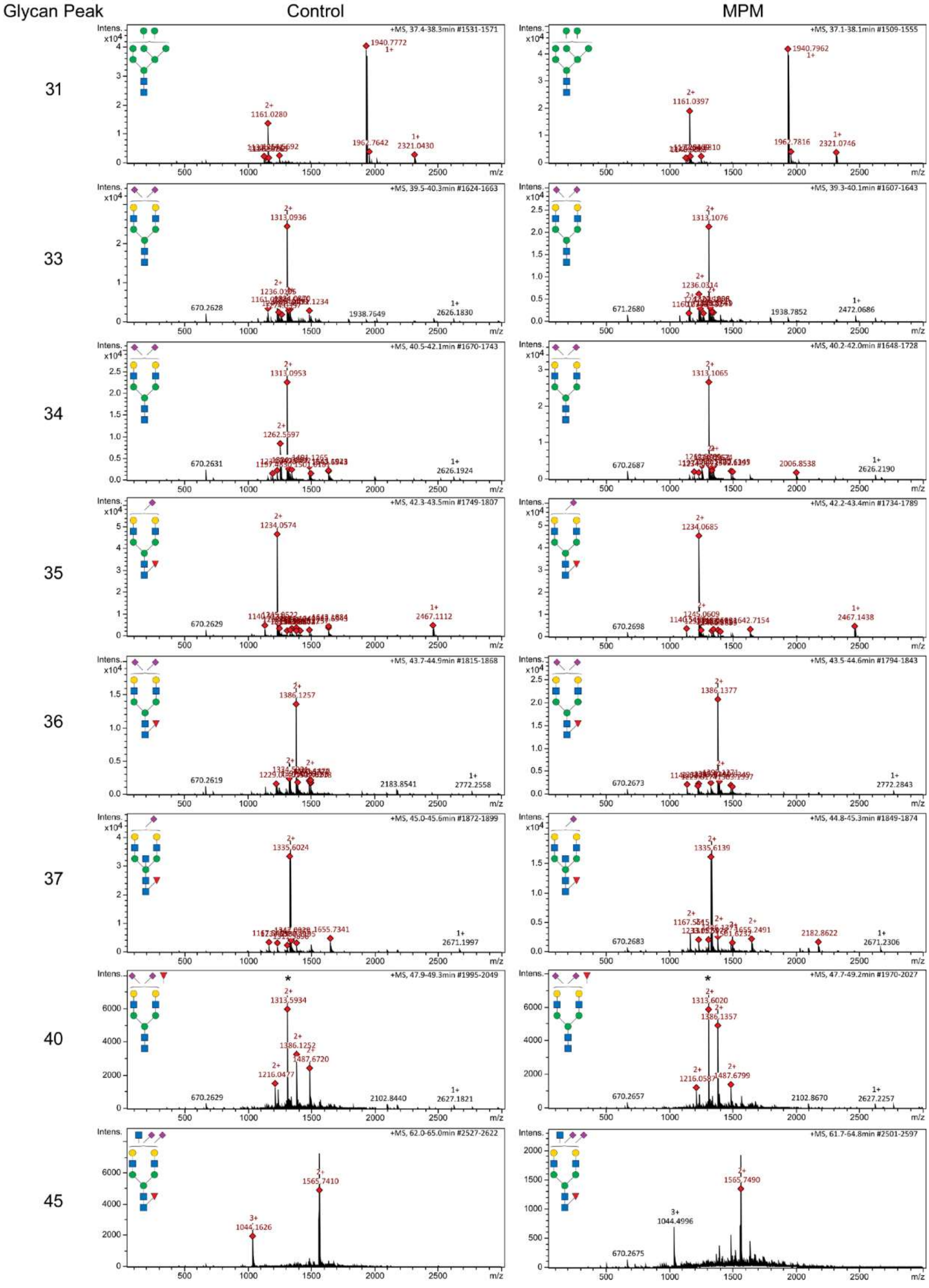

Glycan Peak
Control
MPM
31
33
34
35
36
37
40
45
Intens.
x10^4
m/z
+MS, 37.4-38.3min #1531-1571
1940.7772
1161.0280
2321.0430
+MS, 37.1-38.1min #1509-1555
1940.7962
1161.0397
2321.0746
+MS, 39.5-40.3min #1624-1663
1313.0936
1236.0325
670.2628
1938.7649
2626.1830
+MS, 39.3-40.1min #1607-1643
1313.1076
1236.0314
671.2680
1938.7852
2472.0686
+MS, 40.5-42.1min #1670-1743
1313.0953
1262.5597
670.2631
2626.1924
+MS, 40.2-42.0min #1648-1728
1313.1065
670.2687
2006.8538
2626.2190
+MS, 42.3-43.5min #1749-1807
1234.0574
670.2629
2467.1112
+MS, 42.2-43.4min #1734-1789
1234.0685
670.2698
1642.7154
2467.1438
+MS, 43.7-44.9min #1815-1868
1386.1257
670.2619
2183.8541
2772.2558
+MS, 43.5-44.6min #1794-1843
1386.1377
670.2673
2772.2843
+MS, 45.0-45.6min #1872-1899
1335.6024
1655.7341
2671.1997
+MS, 44.8-45.3min #1849-1874
1335.6139
670.2683
2182.8622
2671.2306
+MS, 47.9-49.3min #1995-2049
1313.5934
1386.1252
1487.6720
1216.0477
670.2629
2102.8440
2627.1821
+MS, 47.7-49.2min #1970-2027
1313.6020
1386.1357
1216.0587
1487.6799
670.2657
2102.8670
2627.2257
+MS, 62.0-65.0min #2527-2622
1565.7410
1044.1626
+MS, 61.7-64.8min #2501-2597
1565.7490
1044.4996
670.2675
