# Supplementary material for: Glycan Fingerprint of Malignant Pleural Mesothelioma
Source: Int J Mol Sci. 2026 Jul 9;27(14):6134. doi: 10.3390/ijms27146134 (PMC13410575; doi:10.3390/ijms27146134)
Supplement: Supplementary file 1 [file ijms-27-06134-s001.zip › S9_CNN_Code_Full.pdf]

```
import pandas as pd
import numpy as np
from sklearn.preprocessing import LabelEncoder, StandardScaler
from sklearn.metrics import confusion_matrix, precision_score, recall_score, ConfusionMatrixDisplay
import matplotlib.pyplot as plt
import torch
import torch.nn as nn
import torch.optim as optim
from torch.utils.data import DataLoader, TensorDataset
from pathlib import Path
import csv

train_file_path = 'DS_MezoPleur_airPls_SNV_Glsw_Train_X.csv'
test_file_path  = 'DS_MezoPleur_airPls_SNV_Glsw_Test_X.csv'

# ================== ONE SPECIFIED RANGE ==================
range_start = 450
range_end   = 3650

train_df = pd.read_csv(train_file_path)
test_df  = pd.read_csv(test_file_path)

# Kolone s valnim brojevima (identične u oba fajla)
wavenumbers = train_df.columns[2:].astype(float)

train_classes_raw = train_df.iloc[:, 1]
test_classes_raw  = test_df.iloc[:, 1]

# ================== CLASS ENCODING==================
label_encoder = LabelEncoder()
y_train = label_encoder.fit_transform(train_classes_raw)
```

```
y_test  = label_encoder.transform(test_classes_raw)



# ================== RANGE VALIDATION ==================
if not (wavenumbers.min() <= range_start < range_end <= wavenumbers.max()):
    raise ValueError(f"Traženi raspon {range_start}-{range_end} je izvan spektra "
                     f"({int(wavenumbers.min())}-{int(wavenumbers.max())}).")

# Filtriraj kolone po rasponu valnih brojeva
valid_columns = (wavenumbers >= range_start) & (wavenumbers <= range_end)
selected_wavenumbers = wavenumbers[valid_columns]
if selected_wavenumbers.size == 0:
    raise ValueError(f"Nema podataka za raspon {range_start}-{range_end}.")

X_train_raw = train_df.iloc[:, 2:].values[:, valid_columns].astype(float)
X_test_raw  = test_df.iloc[:, 2:].values[:, valid_columns].astype(float)

# ================== STANDARDIZATION (fit on train) ==================
scaler = StandardScaler()
X_train_scaled = scaler.fit_transform(X_train_raw)
X_test_scaled  = scaler.transform(X_test_raw)

# ================== CNN INPUT SHAPE ==================
X_train_reshaped = X_train_scaled.reshape(-1, 1, 1, X_train_scaled.shape[1])
X_test_reshaped  = X_test_scaled.reshape(-1, 1, 1, X_test_scaled.shape[1])

# ================== TORCH DATASETI/DATALOADERI ==================
X_train_tensor = torch.tensor(X_train_reshaped, dtype=torch.float32)
y_train_tensor = torch.tensor(y_train, dtype=torch.long)
X_test_tensor  = torch.tensor(X_test_reshaped, dtype=torch.float32)
y_test_tensor  = torch.tensor(y_test, dtype=torch.long)
```

```
train_dataset = TensorDataset(X_train_tensor, y_train_tensor)
test_dataset  = TensorDataset(X_test_tensor, y_test_tensor)

train_loader = DataLoader(train_dataset, batch_size=32, shuffle=True)
test_loader  = DataLoader(test_dataset, batch_size=32, shuffle=False)

# ================= CNN MODEL  ======================
class CNN(nn.Module):
    def __init__(self, input_size, num_classes):
        super().__init__()
        self.conv1 = nn.Conv2d(1, 16, kernel_size=(1, 3), padding=(0, 1))
        self.pool  = nn.MaxPool2d(kernel_size=(1, 2))
        self.conv2 = nn.Conv2d(16, 32, kernel_size=(1, 3), padding=(0, 1))
        with torch.no_grad():
            dummy = torch.zeros(1, 1, 1, input_size)
            out = self.pool(torch.relu(self.conv1(dummy)))
            out = self.pool(torch.relu(self.conv2(out)))
            self.flattened = out.view(1, -1).size(1)
        self.fc1 = nn.Linear(self.flattened, 128)
        self.fc2 = nn.Linear(128, 64)
        self.fc3 = nn.Linear(64, num_classes)

    def forward(self, x):
        x = self.pool(torch.relu(self.conv1(x)))
        x = self.pool(torch.relu(self.conv2(x)))
        x = x.view(x.size(0), -1)
        x = torch.relu(self.fc1(x))
        x = torch.relu(self.fc2(x))
        x = self.fc3(x)
        return x
```

```python
# ================== MODEL/OPTIMIZER INITIALIZATION ==================

input_size = X_train_scaled.shape[1]
num_classes = len(np.unique(y_train))
model = CNN(input_size=input_size, num_classes=num_classes)
criterion = nn.CrossEntropyLoss()
optimizer = optim.Adam(model.parameters(), lr=0.001)

# ================== TRAINING ==================
epochs = 50
for epoch in range(epochs):
    model.train()
    for inputs, labels in train_loader:
        optimizer.zero_grad()
        outputs = model(inputs)
        loss = criterion(outputs, labels)
        loss.backward()
        optimizer.step()

# ================== EVALUATION ==================
model.eval()
correct = 0
total = 0
predicted_classes = []
actual_classes = []
with torch.no_grad():
    for inputs, labels in test_loader:
        outputs = model(inputs)
        _, predicted = torch.max(outputs, 1)
        total += labels.size(0)
```

```
        correct += (predicted == labels).sum().item()
        predicted_classes.extend(predicted.cpu().numpy())
        actual_classes.extend(labels.cpu().numpy())

test_accuracy = correct / total if total > 0 else 0.0

# ================== CONFUSION MATRIX ==================
cm = confusion_matrix(actual_classes, predicted_classes, labels=np.arange(num_classes))
precision = precision_score(actual_classes, predicted_classes, average="weighted", zero_division=0)
recall = recall_score(actual_classes, predicted_classes, average="weighted", zero_division=0)

column_sums = np.sum(cm, axis=0)
with np.errstate(divide='ignore', invalid='ignore'):
    specificities = np.diag(cm) / column_sums
    specificities[np.isnan(specificities)] = 0
specificity = np.mean(specificities)

cm_dir = Path("confusion_matrices_mzpl")
cm_dir.mkdir(parents=True, exist_ok=True)
cm_path = cm_dir / f"cm_{int(range_start)}-{int(range_end)}.png"

fig, ax = plt.subplots()
disp = ConfusionMatrixDisplay(confusion_matrix=cm, display_labels=label_encoder.classes_)
disp.plot(cmap=plt.cm.Blues, xticks_rotation='vertical', colorbar=False, ax=ax)
ax.set_title(f"Matrica zabune: {range_start}-{range_end} cm⁻¹")
fig.tight_layout()
fig.savefig(cm_path, dpi=300, bbox_inches='tight')
plt.close(fig)

range_tag = f"{int(range_start)}-{int(range_end)}"
print("\nSummary of Results:")
```

```
print("Range, Accuracy, Precision, Recall, Specificity")
print(f"{range_tag}, {test_accuracy:.4f}, {precision:.4f}, {recall:.4f}, {specificity:.4f}")

output_file = f"mezoteliomipleure_{range_tag}.csv"
with open(output_file, mode="w", newline="") as file:
    writer = csv.writer(file)
    writer.writerow(["Range", "Accuracy", "Precision", "Recall", "Specificity"])
    writer.writerow([
        range_tag,
        f"{test_accuracy:.4f}",
        f"{precision:.4f}",
        f"{recall:.4f}",
        f"{specificity:.4f}"
    ])

print(f"\nSažetak spremljen u: {output_file}")
print(f"Slika matrice zabune: {cm_path}")
```
